# Unpruning improvement the quality of tea through increasing the levels of amino acids and reducing contents of flavonoids and caffeine

**DOI:** 10.3389/fnut.2022.1017693

**Published:** 2022-09-29

**Authors:** Ruoyu Li, Kunyi Liu, Zhengwei Liang, Hui Luo, Teng Wang, Jiangshan An, Qi Wang, Xuedan Li, Yanhui Guan, Yanqin Xiao, Caiyou Lv, Ming Zhao

**Affiliations:** ^1^College of Tea Science and College of Food Science and Technology and College of Agronomy and Biotechnology, Yunnan Agricultural University, Kunming, China; ^2^The Key Laboratory of Medicinal Plant Biology of Yunnan Province and National and Local Joint Engineering Research Center on Germplasm Innovation and Utilization of Chinese Medicinal Materials in Southwestern China, Yunnan Agricultural University, Kunming, China; ^3^College of Wuliangye Technology and Food Engineering, Yibin Vocational and Technical College, Yibin, China

**Keywords:** pruning, *Camellia sinensis*, sun-dried green tea, metabolomics analysis, sensory characteristic

## Abstract

Tea tree [*Camellia sinensis* var. *sinensis* or *assamica* (L.) O. Kuntze], an important crop worldwide, is usually pruned to heights of 70 to 80 cm, forming pruned tea tree (PTT) plantations. Currently, PTTs are transformed into unpruned tea tree (UPTT) plantations in Yunnan, China. This has improved the quality of tea products, but the underlying reasons have not been evaluated scientifically. Here, 12 samples of sun-dried green teas were manufactured using fresh leaves from an UPTT and the corresponding PTT. Using sensory evaluation, it was found that the change reduced the bitterness and astringency, while increasing sweetness and umami. Using high performance liquid chromatography detection showed that the contents of free amino acids (theanine, histidine, isoleucine and phenylalanine) and catechin gallate increased significantly (*P* < 0.05), whereas the content of alanine decreased significantly (*P* < 0.05). A liquid chromatography–mass spectrometry-based metabolomics analysis showed that the transformation to UPTT significantly decreased the relative levels of the majority of flavonols and tannins (*P* < 0.05), as well as γ-aminobutyric acid, caffeine and catechin (epigallocatechin, catechin, epigallocatechin gallate, gallocatechin gallate), while it significantly increased the relative contents of catechins (gallocatechin, epicatechin, epicatechin gallate and catechin gallate), phenolic acids and some amino acids (serine, oxidized glutathione, histidine, aspartic acid, glutamine, lysine, tryptophan, tyramine, pipecolic acid, and theanine) (*P* < 0.05). In summary, after transforming to UPTT, levels of amino acids, such as theanine increased significantly (*P* < 0.05), which enhanced the umami and sweetness of tea infusions, while the flavonoids (such as kaempferol, myricetin and glycosylated quercetin), and caffeine contents decreased significantly (*P* < 0.05), resulting in a reduction in the bitterness and astringency of tea infusions and an increase in tea quality.

## Introduction

Tea tree [*Camellia sinensis* var. *sinensis*, or var. *assamica* (L.) O. Kuntze] is an important crop for manufacturing tea, which is the most consumed beverage after water (1, 2). According the World Food and Agriculture Organization, the world’s tea planting area was 8,264,698 hectares and distributed among 48 countries in 2019, with 3,185,311 hectares being in China, accounting for 38.5% of the global area (3). To improve production and plucking efficiency, tea trees are generally pruned to a height of 70 to 80 cm, forming pruned tea tree (PTT) plantations, which are dominant both worldwide and in China (4, 5). However, there are also unpruned tea tree (UPTT) plantations in China, especial in Yunnan Province, which has 44,666.67 hectares of UPTT plantations (3).

**FIGURE 1:**
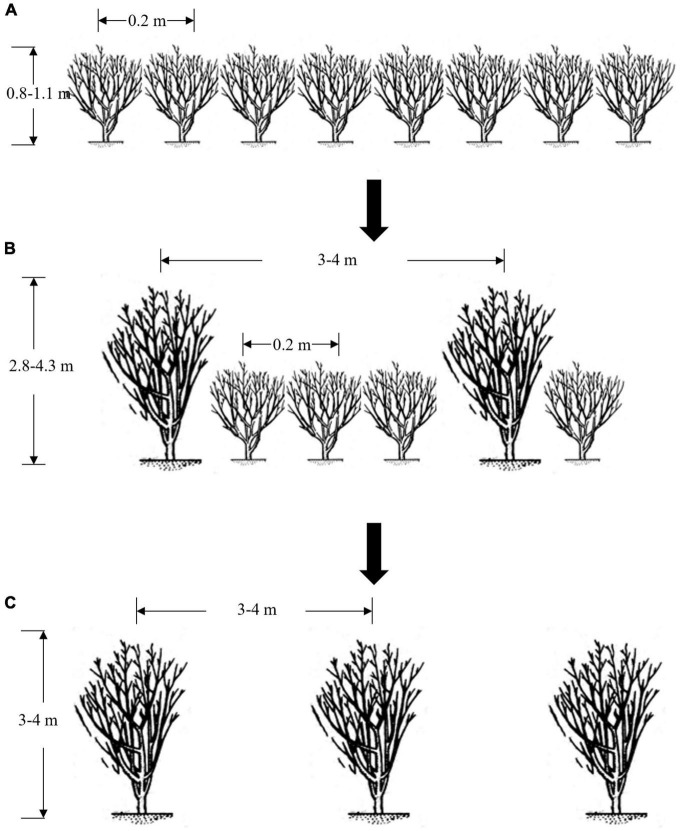
Scheme for the transformation of a pruned to unpruned tea tree plantation. Tea tree plantations that are pruned **(A)**; transforming **(B)**; unpruned **(C)**.

Fresh leaves of both PTT and UPTT are usually used to manufacture sun-dried green tea in Yunnan Province, and then, they are further manufactured into pu-erh tea by microbial fermentation or compression (6, 7). In general, the sensory quality of pu-erh tea manufactured from UPTT is considered better than that of PTT, resulting in the price of the former being higher (8). Because of the higher financial returns, many PTT plantations are being transformed to UPTT plantations using the following process: main branches of one tree were left unpruned and grew to arbor after 5–8 years (Figure 1A). Then, other PTTs were felled, and the UPTTs were reserved, forming UPTT plantations (Figures 1B,C). After transforming into UPTT plantations, the yields decreased; however, the profits increased owing to the higher prices of the manufactured tea products. Therefore, the transformation of PTTs to UPTTs became an interesting cultivation trend in Yunnan, and there were 4,833 hm^2^ [Ning’er County (8), Huilian Base (9), Muga Township (10), Jiangcheng Township (11), and Mojiang Township (12)] and 1,400 hm^2^ [Jingmai Mountain (13) and Dadugang Base (14)] of transformed plantations in Pu’er and Jinghong cities, respectively, Yunnan Province, China.

Pruning is an important cultivation management technique used in tea cultivation that influences the chemical compounds, in fresh tea leaves and tea quality (15). It can affect the regulation of flavonoid biosynthesis, phenylpropanoid biosynthesis and amino acid metabolism (16). For example, the epigallocatechin gallate (EGCG) contents in tea manufactured from PTTs are significantly higher than in those from UPTTs (17). Additionally, the free amino acids, as well as the theanine, chlorophyll and floral-honey aromatic substance, levels in tea leaves of UPTTs are higher than those of PTTs, whereas the catechins levels are lower (18). Previously, we showed that the contents of amino acids in sun-dried green tea manufactured from UPTT increased, whereas the level of polyphenols and bitterness decreased, which suggested that the transformation of PTTs to UPTTs improves the quality of tea products (4). However, what is responsible for the improved sensory quality remains unknown and warrants systematically evaluation.

To survey what occurs during the change from PTT to UPPT, 12 samples of sun-dried green teas were manufactured from fresh leaves of UPTTs and their corresponding PTTs. Then, the differences in sensory qualities and chemical compositions were investigated through sensory evaluation, high-performance liquid chromatography (HPLC) assays and ultra-performance liquid chromatography–tandem mass spectrometry (UPLC–MS/MS)-based metabolomics analyses.

## Materials and methods

### Chemical standards

Reference standards (> 98% purity) for 12 compounds, gallic acid, (−)-gallocatechin, (−)-epigallocatechin, (−)-catechin, caffeine, EGCG, (−)-epicatechin, (−)-gallocatechin gallate, (−)-epicatechin gallate, taxifolin, (−)-catechin gallate, rutin, ellagic acid, myricetin, quercetin, luteolin and kaempferol, were obtained from Chengdu Must Biotechnology (Chengdu, China). Reference standards (>98% purity) for 16 amino acids, aspartic acid, glutamic acid, serine, histidine, glycine, threonine, arginine, alanine, tyrosine, cysteine, methionine, phenylalanine, isoleucine, leucine, lysine and proline, were purchased from Agilent Technologies (Beijing, China). Reference standards (>98% purity) of theanine and γ-aminobutyric acid were purchased from the National Institutes for Food and Drug Control (Beijing, China). Acetonitrile and methanol for the HPLC analysis were purchased from Beijing Mirida Technology Co., Ltd., (Beijing, China). All the chemicals and reagents were of analytical grade. The concentrations of the 2 alkaloids and 16 polyphenols in tea leaves were determined using an Agilent 1200 series HPLC system having a Poroshell 120 EC-C18 column (4.6 × 100 mm, 2.7 μm) fitted with a C18 guard column (Agilent Technologies, Santa Clara, CA, USA). The detailed elution conditions have been described previously (19). The contents of 18 amino acids in tea leaves were measured in accordance with previous reports (20, 21). Each sample was analyzed with three repetitions.

### Manufacturing of sun-dried green tea

Tea plantations were located at 22°53′48″N 101°18′47″E (P1) and 23°3′27″N 100°56′60″E (P2) in Pu’er City. These PTT plantations were developed in 1980. In 2010, these plantations began the transformation into UPTT plantations, and the UPTTs has been unpruned while PTTs did not for 11 years. Thus, there are newly transformed UPTTs and corresponding PTTs on the same plantation. Fresh tea samples with one bud and two leaves were plucked from both newly transformed UPTTs and their corresponding PTTs, in 2020. Then, divide these fresh tea leaves into 12 samples, a total of 180 kilograms, were manufactured into sun-dried green tea leaves in accordance with the general technology of the YUNNAN Pu-erh Tea Group, which is briefly as follows: (1) fresh tea leaves were plucked and taken to the factory; (2) fresh leaves were fixed manually using electric pans; (3) fixed tea leaves were rolled and dried under sun-light to a moisture content of less than 10%. A total of 12 sun-dried green tea samples were collected.

### Sensory evaluation

The sensory evaluation was performed in accordance with Chinese standard (GB/T 23776-2018) (22). Briefly, 3 g of dried tea leaves was infused in 150 ml boiled water for 5 min, and then, the aroma, taste and color of the tea infusion were evaluated by nine trained sensory testers. A quantitative descriptive analysis was performed to evaluate the flavor profiles, including bitterness, astringency, sweetness, thickness and umami (23). The team members used a night-point scale, ranging from 0 (absent) to 9 (extremely strong), to assess the intensity levels of the taste attributes.

### Metabolomics analysis

The 12 samples were sent to the Metware Company to develop metabolomics experiments using UPLC (Shim-Pack CBM30A, Shimadzu, Kyoto, Japan) and MS/MS (QTRAP 4500, Applied Biosystems, Foster City, CA, USA).^1^ Each sample was placed in a vacuum freeze-drying machine (Scientz-100F, Ningbo, China) and ground (30 Hz for 1.5 min) to a powder in a grinding machine (MM 400, Retsch, Germany). In total, 100 mg powder was weighed and dissolved in 0.6 ml 70% methanol extract. The dissolved sample was vortexed six times and then placed at 4°C overnight in a refrigerator to improve the extraction rate. After centrifugation (10,000 *g* for 10 min), the supernatant was extracted, filtered through a 0.22-μm membrane and stored in the injection bottle. Each extract was separated on an ACQUITY UPLC HSS T3 C18 column (2.1 × 100 mm, 1.8 μm; Waters Corporation, Milford, MA, USA). The mobile phases A and B were ultrapure water with 0.04% acetic acid and acetonitrile with 0.04% acetic acid, respectively. The elution gradient was as follows: 0.00 min, the proportion of B phase was 5%; 0–10.00 min, the proportion of B phase linearly increased to 95%; 10–11.00 min, the proportion of B phase was maintained at 95%; 11–12.00, the proportion of B phase decreased to 5%; and 12–14.00 min, the proportion of B phase was maintained at 5% for balance. The flow rate was set at 0.35 ml/min, and the column temperature was 40°C. A 4-μl sample was injected. The temperature of the electrospray ionization (ESI) was maintained at 550°C, the MS voltage was maintained at 5,500 V, the curtain gas was 30 psi, and the collision-activated dissociation was set at maximum. In triple quadrupole MS (QQQ), each ion pair is scanned according to the optimized declustering potential and collision energy. The quantification of metabolites was performed using multiple reaction monitoring mode of QQQ (24).

Data filtering, peak detection, alignment and calculations were performed using Analyst 1.6.3 software (AB Sciex). Based on the self-built database MWDB (Metware database), the material was qualitatively analyzed using the secondary spectral information. Isotope signals, repetitive signals containing K^+^, Na^+^, and NH_4_^+^ ions, and repetitive signals of fragments of other larger molecular weight substances were removed. The characteristic ions of each substance were screened using QQQ, and the signal intensity levels of the characteristic ions were obtained in the detector. The offline mass spectral file of the sample was opened using MultiQuant software, and the integration and correction of chromatographic peaks were performed. The area of each chromatographic peak represents the relative content of the corresponding substance. Finally, all the chromatographic peak area integration data were saved.

### Statistical analysis

Comparison groups were subjected to principal component analyses (PCAs) and (orthogonal) partial least-squares-discriminant analyses (OPLS-DAs) using MetaboAnalyst.^2^ Variable importance in the projection (VIP) was used to rank the overall contribution of each variable in the OPLS-DA model, and variables with VIP > 1.0, *P* < 0.05, and fold change FC > 1.5 or FC < 0.5 were considered differentially changed metabolites (25). One-way analyses of variance and Pearson’s correlation analyses were calculated using SPSS 26.0 (SPSS Inc., Chicago, IL, USA).

## Results

### Difference of sensory quality

**FIGURE 2:**
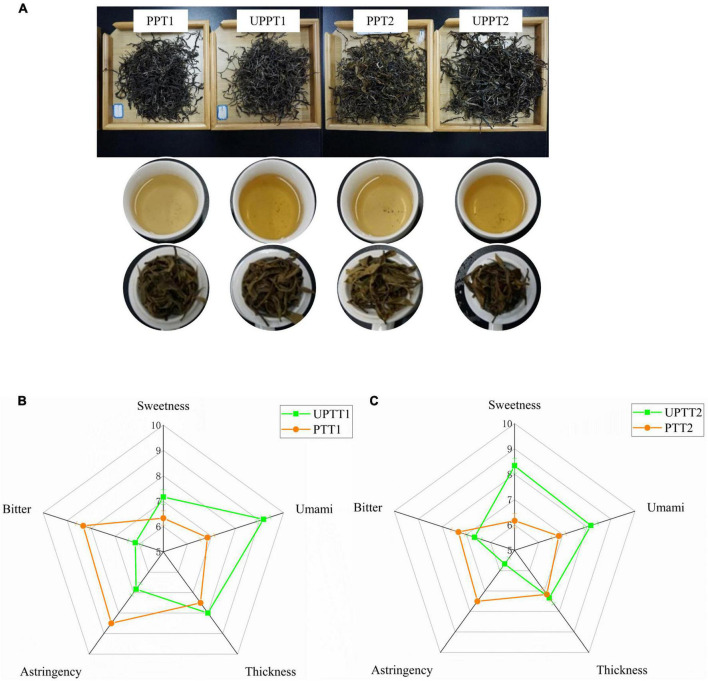
The infusion colors **(A)** and tastes **(B,C)** of sun-dried green tea manufactured from fresh tea leaves of newly transformed unpruned tea tree (UPTT) and their corresponding pruned tea tree (PTT).

Four sun-dried green teas, with triple repetitions, were manufactured from fresh tea leaves of newly transformed UPTTs and their corresponding PTTs. The sensory characteristics of infused tea leaves of sun-dried green tea manufactured in this work were similar to those of normal products (26), such as the color of infusions being yellow (Figure 2A). However, the tastes were slightly different. The bitterness [(7.33 ± 0.29) – (8.33 ± 0.53)] and astringency (8.06 ± 0.48) of tea manufactured from UPTTs were significantly reduced (*P* < 0.05), and the sweetness [(7.17 ± 0.72) – (8.33 ± 0.29)] and umami [(8.17 ± 0.30) – (9.17 ± 0.29)] were significantly increased (*P* < 0.05) (Figures 2B,C). This validated that the transformation of PTT to UPTT improved the sensory quality of sun-dried green tea (23). Consequently, we further surveyed the differences in chemical compounds between the two types of tea.

### Differences in the characteristic chemical compounds of tea

**FIGURE 3:**
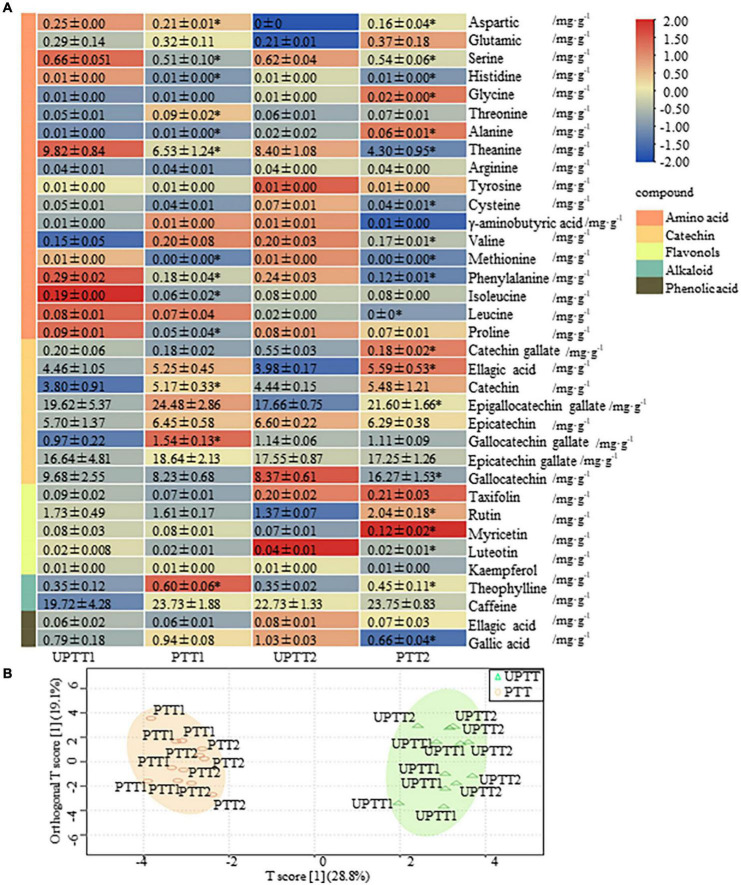
Levels of 36 characteristic chemical compounds (amino acids, catechins, flavonols, alkaloids, and phenolic acid) in tea leaves. OPLS-DA-based levels of 36 characteristic chemical compounds in pruned tea tree (PTT) and unpruned tea tree (UPTT) (unit: mg/g) **(A)**; Compounds detected by high-performance liquid chromatography (HPLC) **(B)**.

To investigate the differences in chemical compounds, 36 characteristic chemical compounds of tea were detected by HPLC, including free amino acids, catechins, flavonols, alkaloids and phenolic acids (Figure 3A). The transformation of PTT to UPTT increased the sum content of free amino acids from (7.22 ± 1.76) to (11.05 ± 1.38) mg/g, as well as the individual contents of six amino acids, cysteine, theanine, phenylalanine, histidine, isoleucine and methionine.

Eight catechins had levels ranging from (0.18 ± 0.02) mg/g to (24.48 ± 2.86) mg/g, with sum contents ranging from (60.68 ± 5.37) mg/g to (71.86 ± 6.50) mg/g in PTTs and UPTTs. The dominate catechin was EGCG, with a content of (17.66 ± 0.75)– (19.62 ± 5.37) mg/g in UPTT and (21.60 ± 2.18)– (24.48 ± 2.86) mg/g in PTT. Both the sum content of catechins and the level of EGCG were higher in PTTs than in UPTTs, which is consistent with previous studies (27).

In this work, five flavonols had levels ranging from (0.01 ± 0.00) to (2.24 ± 0.30) mg/g, with sum contents of (1.81 ± 0.36) to (2.09 ± 0.37) mg/g. Rutin had the highest level in each group, with contents of (1.61 ± 0.17)– (2.04 ± 0.18) mg/g and (1.37 ± 0.07)– (2.24 ± 0.30) mg/g in sun-dried green tea produced from PTT and UPTT, respectively. Flavonols are responsible for the most significant flavors in tea, forming compounds that result in the tastes of astringency and bitterness (28). The total flavonols content of PTT was higher than that of UPTT, which may result in the decreased astringency and bitterness. CA is the main methylxanthine component among the tea alkaloids, and it is an important quality factor owing to its psychoactive effects and distinctive bitter taste (29). In this work, two alkaloids had levels ranging from (0.35 ± 0.02) to (29.92 ± 3.55) mg/g, and sum contents ranged from (22.83 ± 2.40) to (25.52 ± 4.29) mg/g. CA was the alkaloid with the highest levels in both UPTTs [(19.72 ± 4.28) mg/g– (23.73 ± 1.88) mg/g] and PTTs [(22.73 ± 1.33) mg/g– (23.75 ± 0.83) mg/g], but the contents were higher in the UPTTs compared with the PTTs. In addition, Theo was decreased in UPTTs (*P* < 0.05).

To gain an overview of the differences in chemical compounds, we further performed an OPLS-DA. In the score, 2 scores accounted for 47.9% of the total variation, and UPTT and PTT formed two clusters, which validated that the levels of tea characteristic chemical compounds after transformation were different (Figure 3B). The sum contents of amino acids and phenolic acids in UPTTs were higher than in PTTs, and the sum contents of catechins, alkaloids and flavonoids in UPTTs were lower than in PTTs, respectively. Contents of theanine, phenylalanine, serine, methionine and histidine in the UPTTs were higher than in the PTTs, but the theophylline content in the UPTTs was lower than in the PTTs. In summary, the contents of flavonoids and CA in PTTs were higher than in UPTTs, whereas the contents of amino acids in UPTTs were higher than in PTTs.

### The differences in metabolites

**FIGURE 4:**
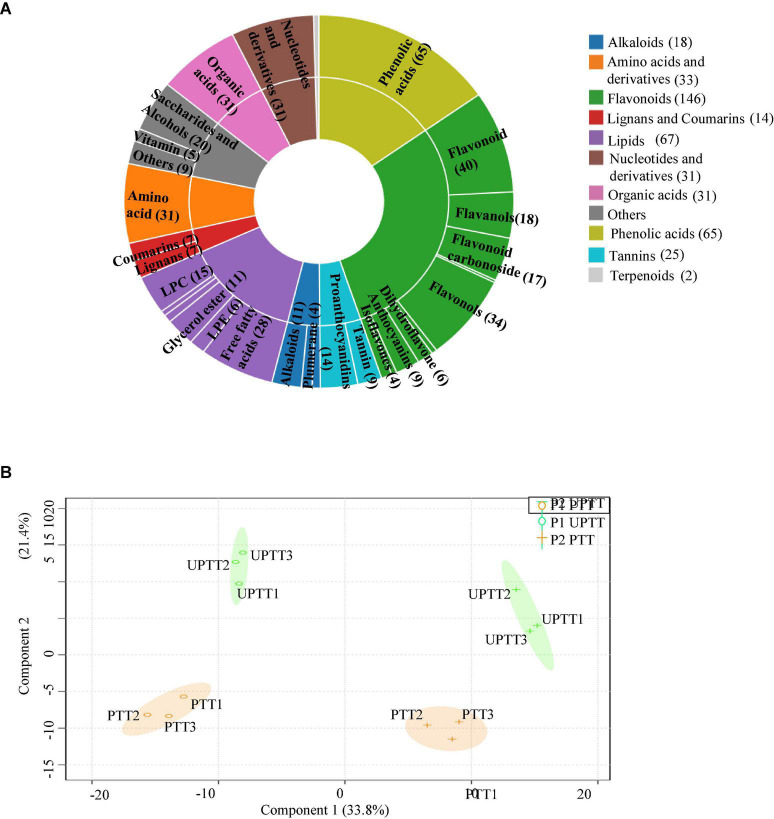
The metabonomics analysis. Classification of identified metabolites **(A)**; OPL-based peak areas of identified metabolites **(B)**.

To further investigate the differences in chemical compounds after transformation (Figure 4B), we further developed a widely targeted metabonomic analysis, and 451 metabolites were identified, including luteolin, kaempferol, tyrosine, tyramine and sorbitol (Figure 4A; Supplementary Information 1, sheet 1). These metabolites were grouped into 11 classes, and major metabolites included flavonoids (146), phenolic acids (65) and lipids (67). These were followed by amino acids and derivatives (33), nucleotides and derivatives (31), organic acids (31), alkaloids (18), lignans and coumarins (14), tannins (10), and terpenoids (2). They were further grouped into 30 sub-classes, including flavonols (34), free fatty acids (28), saccharides and alcohols (20), flavanols (18), flavonoid carbonoside (17), Lysophosphatidylcholine (15), proanthocyanidins (14), anthocyanins (9), and isoflavones (4).

**FIGURE 5:**
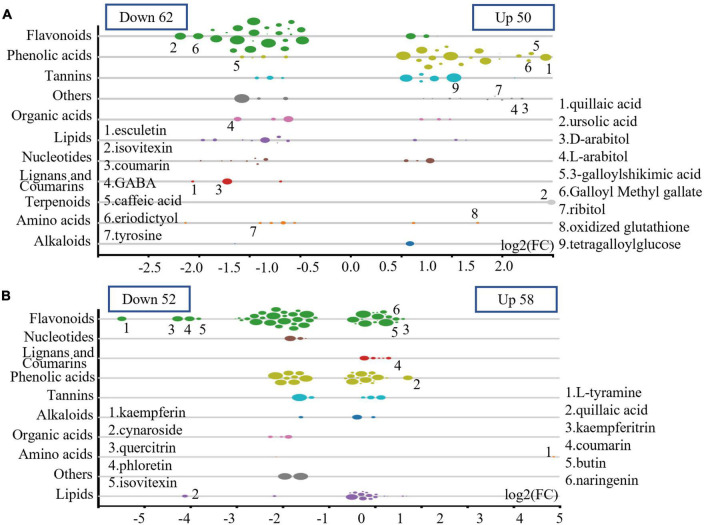
Differentially changed metabolites in P1 **(A)** and P2 **(B)**.

In plantation P1, the relative levels (RLs) of 62 metabolites in UPTTs were lower than in PTTs (VIP > 1, FC < 0.66 and *P* < 0.05), and the majority of these metabolites were flavonoids (24), followed by lipids (8), nucleotides and derivatives (7), amino acids and derivatives (5), phenolic acids (4) and lignans and coumarins (3) and tannins (3), including esculetin, isovitexin, coumarin, γ-aminobutyric acid, caffeic acid, eriodictyol and tyrosine. In addition, the RLs of 50 metabolites in UPTTs were higher than in PTTs (VIP > 1, FC > 1.5, and *P* < 0.05), and the majority of these metabolites were phenolic acids (19), followed by saccharides and alcohols (8), tannins (6), flavonoids (3), lipids (3) and amino acids and derivatives (2), including quillaic acid, ursolic acid, arabitol, arabitol, 3-galloylshikimic acid, sorbitol, ribitol, oxidized glutathione and xylitol (Figure 5A). The high levels of theanine in UPTTs were in accordance with HPLC measurements.

In plantation P2, the RLs of 52 metabolites in UPTTs were lower than in PTTs (VIP > 1, FC < 0.66, and *P* < 0.05), and the majority of these metabolites were flavonoids (28), followed by phenolic acids (8) organic acids (4) and nucleotides and derivatives (3), including kaempferin, cynaroside, quercitrin, phloretin, and isovitexin. In addition, the RLs of 58 metabolites in UPTTs were higher than in PTTs, and the majority of these metabolites were flavonoids (17), lipids (15) and phenolic acids (15), followed by lignans and coumarins (5) and tannins (3 metabolites), including tyramine, quillaic acid, kaempferitrin, coumarin, rutin and naringenin (VIP > 1, FC > 1.5, and *P* < 0.05) (Figure 5B). The high levels of serine and phenylalanine in UPTTs were in accordance with HPLC measurements.

**FIGURE 6:**
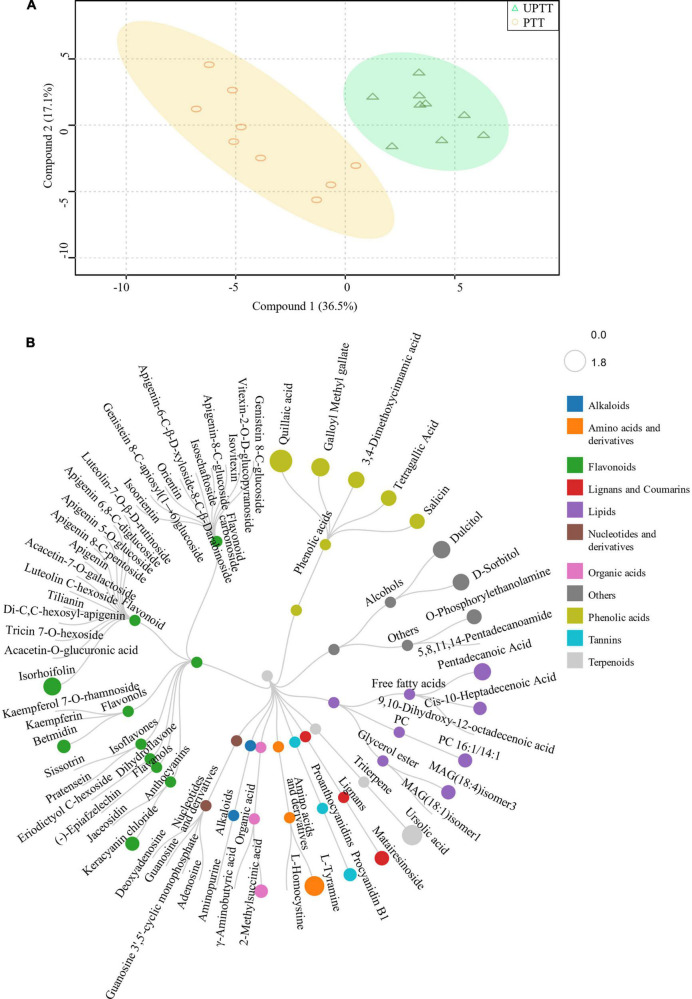
The OPLS-DA analysis of peak areas of identified metabolites **(A)** and differentially changed metabolites **(B)**.

To gain an overview of metabolites differences between UPTTs and PTTs, we developed a new OPLS-DA of mixed data (Figure 6A). The RLs of 20 metabolites, including 5 phenolic acids (salicin, tetragallic acid, 3,4-dimethoxycinnamic acid, galloyl methyl gallate and quillaic acid), 4 lipids (*cis-*10-heptadecenoic acid, PC 16:1/14:1, MAG (18:4) and pentadecanoic acid) and 3 flavonoids (betmidin, keracyanin chloride and isorhoifolin), as well as others, were abundant in UPTTs. In addition, the RLs of 37 metabolites were significantly lower (VIP > 1.0, FC < 0.66, and *P* < 0.05) (Figure 6B), including 27 flavonoids, such as (−)-epiafzelechin, apigenin, sissotrin, orientin, isoorientin, jaceosidin, and pratensein, furthermore, kaempferol 7-O-rhamnoside, kaempferin, genistein 8-C-glucoside, luteolin, vitexin-2-O-D-glucopyranoside, isovitexin, apigenin 6,8-C-diglucoside, apigenin 5-O-glucoside, apigenin-8-C-glucoside, eriodictyol C-hexoside and apigenin 8-C-pentoside, which were more than twofold greater in PTTs compared with UPTTs (Figure 6B). The high levels of epicatechin, epigallocatechin, catechin, γ-aminobutyric acid, luteolin and kaempferol in PTTs were in accordance with HPLC measurements.

### Correlation analysis between sensory evaluation and differential metabolites

**FIGURE 7:**
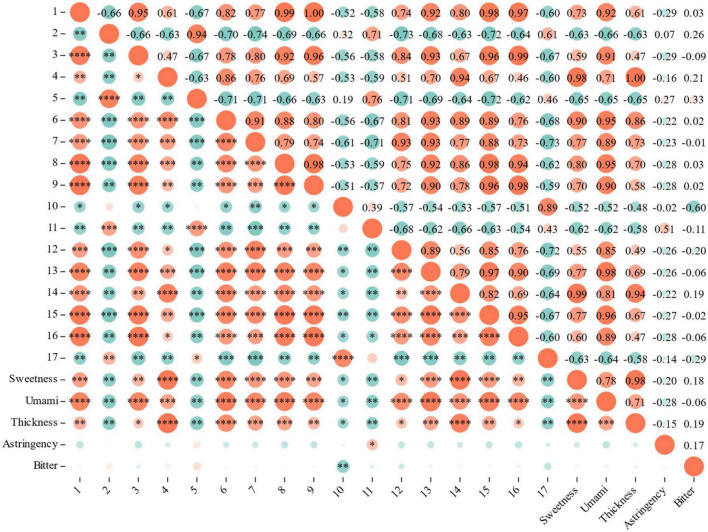
Correlation analysis between sensory evaluation and differential metabolites.

Using Correlation analysis between sensory evaluation and differential metabolites, the RLs of 12 metabolites associated with sweetness, umami and thickness (correlation coefficient > 0.80, *P* < 0.05): 3,4-Dimethoxycinnamic acid, pentadecanoic acid and procyanidin B1 are associated with sweetness, umami and thickness; Betmidin, matairesinoside and quillaic acid associated with sweetness and umami; Tetragallic acid, 2-methylsuccinic acid, sorbitol, dulcitol and salicin are associated with umami; Keracyanin chloride is associated with sweetness (Figure 7).

### Change of metabolism

**FIGURE 8:**
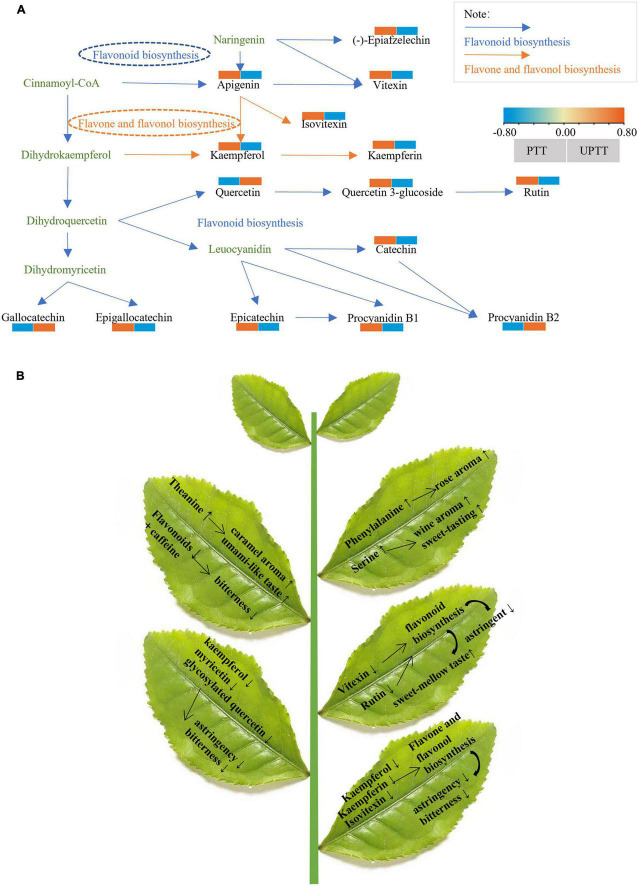
Reconstruction of the flavonoid biosynthetic pathway **(A)**; Schematic diagram of changes in metabolism after unpruning **(B)**.

The different metabolites (VIP > 1, FC > 1.5, and *P* < 0.05) in UPTT and PTT were mapped to the KEGG. The compounds with high content in PTT are enriched to flavone and flavonol biosynthesis, purine metabolism, flavonoid biosynthesis; while, the compounds with high content in UPTT are enriched to isoquinoline alkaloid biosynthesis and tyrosine metabolism. Meanwhile, four metabolic pathways (flavonoid biosynthesis, flavone and flavonol biosynthesis, purine metabolism and tyrosine metabolism) were chosen as key metabolites to characterize the conversion of the main active components of UPTT and PTT (*P* < 0.05) (Figure 8A).

Flavone and flavonol biosynthesis, flavonoid biosynthesis, purine metabolism and their secondary metabolites played an important role in tea quality (30, 31). Because flavonoids levels were significantly changed, we reconstructed the flavonoid biosynthetic pathway that included 14 metabolites, 4 catechins (epicatechin, epigallocatechin, gallocatechin, and catechin), 8 flavonol and flavonol/flavone/flavonoid glycosides (rutin, quercetin, epiafzelechin, kaempferol, kaempferin, quercetin 3-O-glucoside, vitexin, apigenin and isovitexin) and 2 dimeric catechins (procyanidins B1 and B2). 11 metabolites enriched in flavonoid biosynthesis, of which 9 metabolites (epicatechin, epigallocatechin, catechin, rutin, epiafzelechin, quercetin 3-O-glucoside, vitexin, apigenin and procyanidins B1) decreased and 3 metabolites (quercetin, gallocatechin, procyanidin B2) increased. This suggested that unpruning decreased flavonoid biosynthesis (Figure 8A).

## Discussion

At present, the quality characteristics and biochemical components of tea plants with pruned and unpruned have been reported. Sun et al. (27) recently reported the differences in the levels of catechins or their derivatives in tea plants with pruned and unpruned, but their studies lack information on other metabolites. Ruble et al. (15) recently reported the changes in metabolites of tea plants with pruned and unpruned in different seasons, but did not analyze the changes in quality characteristics caused by changes in metabolites. Therefore, we studied the changes of comprehensive metabolites collected from pruned and unpruned tea trees in the same tea garden and their effects on quality characteristics, so as to better understand the metabolic physiology and quality characteristics of unpruned tea cultivation.

### Sensory characteristic in tea leaf metabolites between pruned and unpruned tea trees

The impacts of pruning on chemical compositions of tea leaves have been investigated. In particular, pruning of tea trees leaves through metabolic approaches have been reported in recent years. In abnormal year of vintage with high rainfall, high synthesis of glucose followed by high accumulations of catechin, including its derivatives, in unpruned tea trees, demonstrated intense active photosynthesis compared to pruned tea trees, indicating different metabolic responses of pruned and unpruned tea trees to similar climatic conditions (15); The findings revealed that EGCG accumulation in pruned tea trees was significantly higher than in unpruned tea trees. Low EGCG accumulation in the leaves of unpruned tea trees. Because of the bitter and astringent taste of EGCG, these results provide a certain understanding to the lower bitterness and astringency in teas from “ancient tea trees” (27).

Interestingly, some of amino acids are important flavor-related amino acids, including theanine, alanine, phenylalanine, serine, methionine and histidine. Theanine forms umami-like taste compounds and generates a pronounced caramel aroma. Alanine and serine form sweet-tasting compounds, whereas histidine forms bitter-tasting compounds. Phenylalanine generates the rose aroma and serine generates wine aromas (32). Catechins, especially EGCG and —epicatechin gallate are the major contributors to the bitterness and astringency (33, 34). EGCG accumulation in pruned tea trees was significantly higher than that in unpruned tea trees. SCPL1A expression (encoding a class of serine carboxypeptidase), which has been reported to have a catalytic ability during EGCG biosynthesis, together with LAR, encoding leucoanthocyanidin reductase, was upregulated in the pruned tea trees (27). Theanine accumulation in unpruned tea trees was significantly higher than that in pruned tea trees. The control of MYB regulators in theanine biosynthesis was further demonstrated theanine accumulation in UPTT is mainly controlled by MYB regulators (35). Anthocyanins are also the characteristic metabolites. Procyanidin B1 and Procyanidin B2 were found to be significantly different in UPTT and PTT. Subsequently, pathway analysis revealed that tea trees might generate anthocyanins and proanthocyanidins through the flavonol and flavone glycosides (36). Galloylated catechins are the main compounds responsible for the silky and astringent mouthfeel, whereas CA and non-galloylated catechins enhance bitterness (37). In summary, theanine provides an umami-like taste and caramel aroma, and phenylalanine provides a rose aroma (32). Sorbitol decreases the bitterness provided by CA (38). Therefore, unpruning increased the amino acid levels, resulting in increased umami and sweetness. The decreased contents of flavonoids reduced the bitterness and astringency of the tea infusions.

### Different metabolic pathways of pruned and unpruned tea trees

Previous studies reported that the detailed insight into the chemical and sensory aspects of aromas and flavors was provided (39). Flavonoid biosynthesis, stilbenoid, diarylheptanoid and gingerol biosynthesis, citrate cycle (TCA cycle), and propanoate metabolism may play important roles in the differences among cultivars. The levels of tea polyphenols, flavonoids and amino acids may impact the sensory properties of teas of different cultivars (40). In the current study, the pathway (tyrosine metabolism) was significantly enriched (41, 42) and involved in the biosynthesis of catechins (41). Tyrosine metabolism also involved in the biosynthesis of catechins (41), which may be a factor of catechin reduction in PTT. These results suggested that the conversion pathways of metabolites between the UPTT and PTT, and that metabolic pathways could explain the differences in the presence of differentially exclusive metabolites during transformation of tree type. The current study indicated that flavonoids were accumulated but some amino acids were degraded in PTT samples. Interestingly, flavone and flavonol biosynthesis showed difference in the intercropping system and monoculture system that flavonoids generate more bitter-tasting (43). The content of kaempferol, kaempferin and isovitexin increased in PTT. It should be presumed that kaempferol was metabolized to kaempferin in UPTT (44) (Figure 8A).

Through sensory evaluations, HPLC assays and a metabolomics analysis, we determined that tea qualities improved after unpruning. The umami and sweetness of tea infusions increased, while the bitterness and astringency decreased. These changes resulted from the unpruning-related decrease in the biosynthesis of flavonoids, which reduced astringency and bitterness, as well as the increases in the contents of amino acids, including theanine, isoleucine, histidine and phenylalanine, which increased the umami, aroma and sweetness (Figure 8B). This study demonstrated the differences in sensory qualities and chemical compositions of sun-dried green teas manufactured from pruned and unpruned tea trees, providing a scientific basis for the future improvement of tea quality through cultivation management.

## Data availability statement

The original contributions presented in this study are included in the article/Supplementary material, further inquiries can be directed to the corresponding authors.

## Author contributions

RL: conceptualization, data curation, formal analysis, methodology, software, and writing – original draft. KL: data curation, formal analysis, software, and writing – review and editing. ZL: data curation, formal analysis, and software. HL, JA, and YX: formal analysis and validation. QW, YG, and XL: resources and validation. CL: funding acquisition and supervision. MZ: conceptualization, funding acquisition, methodology, validation, supervision, and writing – review and editing. All authors contributed to the article and approved the submitted version.

## Footnotes

1
https://www.metware.cn/

2
https://www.metaboanalyst.ca/
