# Supplementary material for: Unpruning improvement the quality of tea through increasing the levels of amino acids and reducing contents of flavonoids and caffeine
Source: Front Nutr. 2022 Sep 29;9:1017693. doi: 10.3389/fnut.2022.1017693 (PMC9558131; doi:10.3389/fnut.2022.1017693)
Supplement: Supplementary file 2 [file Data_Sheet_1.PDF]

			log2(FC)	P
1	2	3	4	5
Aminopurine	Alkaloids	Alkaloids	−0. 60695	2. 9054
L-Homocystine	nino acids and derivati	acids and deri	−0. 83938	2. 4475
L-(-)-Tyrosine	nino acids and derivati	acids and deri	−0. 61522	1. 2644
L-Tyramine	nino acids and derivati	acids and deri	0. 64741	1. 011
-5-methoxybenzoyl)3,3',4',5,5',7-	Flavonoids	Flavanols	−1. 9241	2. 0018
van; (2R,3R)-form, 3-O-(3,4-Dih	Flavonoids	Flavanols	−1. 7359	2. 5113
Kaempferol 7-O-rhamnoside	Flavonoids	Flavonols	−1. 4182	1. 5174
Luteolin	Flavonoids	Flavonoid	−1. 3593	1. 5995
kaempferol	Flavonoids	Flavonols	−1. 3421	1. 5006
Genistein 8-C-glucoside	Flavonoids	onoid carbonc	−1. 3241	2. 631
Quercitrin	Flavonoids	Flavonols	−1. 2439	1. 3034
Vitexin-2-O-D-glucopyranoside	Flavonoids	onoid carbonc	−1. 2106	4. 2658
Luteolin-7-O-β-D-rutinoside	Flavonoids	Flavonoid	−1. 1019	3. 371
Isovitexin	Flavonoids	onoid carbonc	−1. 0623	2. 52
Apigenin 5-O-glucoside	Flavonoids	Flavonoid	−1. 0309	2. 1594
Apigenin 6,8-C-diglucoside	Flavonoids	Flavonoid	−0. 9873	4. 4159
Apigenin-8-C-glucoside	Flavonoids	onoid carbonc	−0. 96532	2. 2065
Luteolin C-hexoside	Flavonoids	Flavonoid	−0. 85233	3. 0943
Apigenin 8-C-pentoside	Flavonoids	Flavonoid	−0. 84522	2. 3293
Eriodictyol C-hexoside	Flavonoids	)ihydroflavon	−0. 84059	1. 9675
Acacetin-7-O-galactoside	Flavonoids	Flavonoid	−0. 80829	2. 485
Orientin	Flavonoids	onoid carbonc	−0. 7889	2. 6795
Sissotrin	Flavonoids	Isoflavones	−0. 78622	2. 2985
lianin(Acacetin-7-O-β-D-glucosi	Flavonoids	Flavonoid	−0. 76076	2. 2575
in-6-C-β-D-xyloside-8-C-β-Darat	Flavonoids	onoid carbonc	−0. 74792	2. 2512
C-Hexosyl-apigenin O-pentoside	Flavonoids	onoid carbonc	−0. 7373	1. 3293
(-)-Epiafzelechin	Flavonoids	Flavanols	−0. 73336	1. 1384
Isoorientin	Flavonoids	onoid carbonc	−0. 68683	2. 0527
Di-C,C-hexosyl-apigenin	Flavonoids	Flavonoid	−0. 6844	1. 5822
Apigenin	Flavonoids	Flavonoid	−0. 66487	1. 6696
Isoschaftoside	Flavonoids	onoid carbonc	−0. 6564	2. 7679
Pratensein	Flavonoids	Isoflavones	−0. 65419	2. 3223
nistein 8-C-apiosyl(1→6)glucosi	Flavonoids	onoid carbonc	−0. 62855	2. 1274
Jaceosidin	Flavonoids	Anthocyanins	−0. 61381	3. 6353
Hispidulin	Flavonoids	Flavonoid	−0. 58873	1. 9054
idin 3-rutinoside(Keracyanin chl	Flavonoids	Anthocyanins	0. 60736	1. 1187
Tricin O-saccharic acid	Flavonoids	Flavonoid	0. 69838	1. 2478
Pinoresinol-Hexose	Lignans and Coumarin	Lignans	0. 58844	1. 6114
Matairesinoside	Lignans and Coumarin	Lignans	0. 63646	3. 5572
Terpineol monoglucoside	Lignans and Coumarin	Lignans	0. 65694	1. 3479
10-Dihydroxy-12-octadecenoic ac	Lipids	Free fatty acid	−0. 619	2. 2713
Deoxyadenosine	icleotides and derivati	tides and deri	−1. 3191	2. 2586
ianosine 3',5'-cyclic monophosph	icleotides and derivati	tides and deri	−0. 64874	2. 7209
Cyclic AMP	icleotides and derivati	tides and deri	−0. 60269	3. 0506

5,8,11,14-Pentadecanoamide	Others	Others	−0. 63651	2. 2452
O-Phosphorylethanolamine	Others	Others	0. 58971	1. 9786
D-Sorbitol	Others	arides and Alc	0. 64821	1. 5921
Dulcitol	Others	arides and Alc	0. 72642	1. 6806
p-Coumaroylferuloyltartaric acid	Phenolic acids	Phenolic acids	−0. 8887	1. 9418
1,2,3,6-Tetragalloyl glucose	Phenolic acids	Phenolic acids	0. 64469	1. 4018
'''-O-galloyl-4''''-O-galloyl-4-O-g	Phenolic acids	Phenolic acids	0. 67144	2. 5549
Galloyl Methyl gallate	Phenolic acids	Phenolic acids	0. 89956	1. 7557
Quillaic acid	Phenolic acids	Phenolic acids	1. 0085	2. 3804
Procyanidin B1	Tannins	oanthocyanidi	0. 58669	2. 7407
Procyanidin B4	Tannins	oanthocyanidi	0. 59757	1. 362
			log2(FC)	P
