# Supplementary material for: Unpruning improvement the quality of tea through increasing the levels of amino acids and reducing contents of flavonoids and caffeine
Source: Front Nutr. 2022 Sep 29;9:1017693. doi: 10.3389/fnut.2022.1017693 (PMC9558131; doi:10.3389/fnut.2022.1017693)
Supplement: Supplementary file 3 [file Data_Sheet_2.PDF]

sample	A_1	A_2	A_3	B_1	B_2	B_3
	1	1	1	2	2	2
Galloylquinic acid	3.54E+06	2.87E+06	2.88E+06	1.70E+06	2.22E+06	1.94E+06
3,3'-Bis(3,4,5-trihydroxybenzoyl) theasinensin C	9.58E+03	1.79E+04	1.35E+04	5.65E+03	1.00E+04	8.51E+03
Theasinensin C	2.15E+06	2.39E+06	2.08E+06	1.68E+06	2.04E+06	1.51E+06
3-O-p-Coumaroylquinic acid	2.56E+06	2.90E+06	2.90E+06	3.67E+06	5.02E+06	3.95E+06
Phloretin-3,5-di-C-glucoside	6.45E+05	5.63E+05	4.69E+05	6.00E+05	7.84E+05	5.99E+05
(+)-Afzelechin-3-O-gallate	7.52E+06	5.62E+06	7.31E+06	7.25E+06	6.75E+06	5.50E+06
Astragalin; 2'',6''-Bis-O-(4-hydroxy-E-cinnamoyl)	7.50E+06	8.86E+06	9.10E+06	5.91E+06	5.54E+06	6.77E+06
Isorhamnetin-3-O-rutinoside	3.45E+05	3.08E+05	3.91E+05	4.11E+05	4.06E+05	4.54E+05
Fer-agmatine	3.29E+06	3.28E+06	2.75E+06	3.54E+06	4.42E+06	3.02E+06
p-Coumaroylferuloyltartaric acid	9.71E+04	7.18E+04	1.24E+05	1.89E+05	9.96E+04	1.33E+05
Isorhamnetin hexose-malonate	9.52E+04	8.52E+04	7.46E+04	1.58E+05	2.23E+05	1.64E+05
3,4-Dicaffeoylquinic acid	4.16E+06	4.82E+06	4.95E+06	5.50E+06	4.95E+06	3.68E+06
Quercetin 3-O-rhanosylgalactoside	1.85E+06	2.17E+06	1.98E+06	2.88E+06	2.07E+06	2.47E+06
13-Oxo-9-hydroxy-10-octadecenoic acid	1.15E+05	1.14E+05	1.13E+05	1.35E+05	1.35E+05	1.49E+05
9,10-Dihydroxy-12-octadecenoic acid	1.13E+06	8.94E+05	1.10E+06	3.06E+06	4.10E+06	2.42E+06
13-Hydroxy-9,11-octadecadienoic acid	1.53E+06	1.63E+06	1.50E+06	1.70E+06	1.74E+06	1.85E+06
9-Hydroxy-10,12-octadecadienoic acid	1.50E+06	1.50E+06	1.36E+06	1.62E+06	1.69E+06	1.69E+06
Pinoresinol-Hexose	2.02E+06	2.02E+06	2.37E+06	2.34E+06	3.03E+06	2.40E+06
Pipecolic acid	5.93E+05	5.47E+05	5.96E+05	4.56E+05	4.02E+05	4.49E+05
1,2-N-Methylpipecolic acid	2.74E+05	2.14E+05	2.53E+05	2.16E+05	1.90E+05	2.45E+05
Octadecenoic amide	5.64E+04	3.83E+04	3.36E+04	3.38E+04	5.62E+04	5.93E+04
Tryptamine	1.51E+05	1.43E+05	1.41E+05	1.22E+05	1.77E+05	1.21E+05

Ferulic acid	1.77E+05	2.26E+05	2.26E+05	4.24E+05	5.19E+05	3.94E+05
Spermine	6.68E+06	6.82E+06	7.08E+06	7.65E+06	7.11E+06	7.22E+06
Gallic acid	3.45E+06	3.62E+06	3.69E+06	3.19E+06	2.92E+06	3.31E+06
Vanillic acid	2.31E+04	2.12E+04	2.33E+04	1.75E+04	2.12E+04	2.74E+04
Epigallocatechin gallate	5.57E+06	5.29E+06	5.53E+06	5.25E+06	4.91E+06	4.38E+06
(-)-Epigallocatechin	3.80E+07	3.33E+07	3.30E+07	3.94E+07	3.41E+07	3.54E+07
Taxifolin	2.27E+05	2.00E+05	2.17E+05	3.08E+05	2.14E+05	3.25E+05
Quercitrin	2.11E+06	8.16E+05	6.65E+05	4.65E+06	3.48E+05	1.54E+06
(+)-Gallocatechin	2.04E+06	1.95E+06	1.89E+06	2.35E+06	2.39E+06	1.53E+06
Catechin	3.15E+07	3.38E+07	3.43E+07	3.49E+07	3.28E+07	3.50E+07
Diosmetin	7.20E+05	3.22E+05	4.33E+05	9.68E+05	1.14E+06	1.06E+06
Rutin	2.40E+07	2.55E+07	1.86E+07	2.42E+07	3.12E+07	2.66E+07
Eriodictyol	3.20E+05	2.89E+05	3.29E+05	6.07E+05	6.52E+05	8.52E+05
Isorhamnetin	3.55E+04	3.16E+04	3.31E+04	5.45E+04	7.57E+04	5.73E+04
Apigenin 5-O-glucoside	1.33E+05	8.23E+04	4.76E+04	1.86E+05	2.25E+05	1.15E+05
Kaempferol 7-O-glucosdie	1.38E+07	1.20E+07	1.30E+07	1.49E+07	1.66E+07	1.55E+07
Coniferyl alcohol	1.01E+05	1.06E+05	1.18E+05	2.20E+05	3.31E+05	2.46E+05
Pinoresinol	2.99E+06	2.84E+06	2.65E+06	4.88E+06	6.09E+06	4.96E+06
Indole-3-carboxaldehyde	1.40E+05	1.44E+05	1.45E+05	1.32E+05	1.56E+05	1.49E+05
Myristic Acid	1.41E+07	1.33E+07	1.15E+07	1.29E+07	1.35E+07	1.37E+07
Nicotinamide	6.89E+06	6.66E+06	6.90E+06	7.48E+06	8.32E+06	7.54E+06
2-Methoxybenzoic acid	7.48E+04	8.33E+04	7.20E+04	4.79E+04	3.20E+04	6.91E+04
Shikimic acid	1.74E+06	1.50E+06	1.39E+06	1.98E+06	1.97E+06	2.03E+06
Cyanidin 3-rutinoside(Keracyanin chloride)	3.43E+05	4.89E+05	4.40E+05	3.56E+05	5.74E+05	3.68E+05
Cyanin chloride	2.44E+06	2.21E+06	2.10E+06	1.74E+06	2.17E+06	1.98E+06

Oenin chloride	2.16E+04	2.85E+04	2.05E+04	1.99E+04	2.14E+04	1.67E+04
Chlorogenic acid methyl ester	4.56E+04	5.80E+04	3.97E+04	7.73E+04	9.22E+04	8.00E+04
Protocatechuic acid	4.45E+06	4.70E+06	5.04E+06	6.12E+06	6.36E+06	6.65E+06
Betaine	1.04E+06	1.24E+06	9.32E+05	1.49E+06	1.30E+06	1.36E+06
Succinic acid	8.19E+06	9.77E+06	9.18E+06	1.05E+07	1.01E+07	1.05E+07
Adipic Acid	4.04E+05	3.92E+05	4.52E+05	4.37E+05	6.25E+05	4.94E+05
Ribitol	2.63E+05	2.58E+05	3.06E+05	1.20E+05	1.07E+05	1.37E+05
D-Sorbitol	1.70E+05	1.62E+05	2.00E+05	7.56E+04	5.75E+04	7.29E+04
L-AsparticAcid	4.22E+06	3.84E+06	4.37E+06	4.19E+06	4.97E+06	4.12E+06
L-Leucine	1.18E+07	1.40E+07	1.18E+07	1.77E+07	1.54E+07	1.52E+07
Riboflavin	8.00E+04	5.97E+04	1.05E+05	1.27E+05	2.44E+05	2.20E+05
Anchoic Acid	3.35E+06	3.40E+06	3.64E+06	3.06E+06	3.61E+06	2.93E+06
Uridine	4.05E+05	2.77E+05	3.00E+05	4.47E+05	8.49E+05	4.93E+05
L-(-)-Tyrosine	2.10E+06	2.49E+06	2.09E+06	4.98E+06	4.77E+06	4.06E+06
L-Histidine	2.28E+05	2.47E+05	3.38E+05	3.45E+05	2.63E+05	3.18E+05
Cytosine	1.05E+05	8.32E+04	9.55E+04	1.26E+05	1.44E+05	1.32E+05
L-Valine	3.65E+07	4.11E+07	3.90E+07	5.21E+07	5.11E+07	4.29E+07
L-Isoleucine	2.79E+07	3.22E+07	2.83E+07	4.01E+07	3.64E+07	3.78E+07
L-PyroglutamicAcid	1.82E+04	1.55E+04	1.89E+04	2.10E+04	1.63E+04	1.86E+04
D-(+)-TrehaloseAnhydrous	8.01E+05	7.49E+05	8.46E+05	1.18E+06	9.55E+05	1.36E+06
L-(-)-Malic acid	2.07E+06	1.62E+06	1.99E+06	2.32E+06	2.33E+06	2.26E+06
Kinic acid	7.56E+06	1.09E+07	7.60E+06	1.52E+07	1.53E+07	1.70E+07
Citric Acid	1.05E+07	6.18E+06	1.15E+07	1.27E+07	1.18E+07	1.28E+07
L-Tryptophan	1.27E+07	1.35E+07	1.21E+07	1.20E+07	1.43E+07	1.29E+07
LysoPE 18:1	2.95E+06	2.89E+06	3.24E+06	4.42E+06	5.19E+06	4.38E+06
DL-P-hydroxyphenyllactic acid	7.25E+04	9.21E+04	7.46E+04	7.67E+04	5.98E+04	6.95E+04

(S)-(-)-2-Hydroxyisocaproic acid	1.19E+05	1.52E+05	1.31E+05	1.24E+05	1.36E+05	1.15E+05
D-Xylonic acid	1.98E+06	1.99E+06	2.12E+06	2.36E+06	2.52E+06	2.04E+06
Gallate catechin gallate	4.78E+06	5.28E+06	4.99E+06	4.83E+06	4.58E+06	3.96E+06
Theophylline	5.06E+04	4.98E+04	6.19E+04	6.62E+04	5.05E+04	6.18E+04
Pentadecanoic Acid	2.65E+04	3.92E+04	2.06E+04	1.70E+04	2.04E+04	2.04E+04
Palmitoleic Acid	1.33E+04	1.04E+04	1.17E+04	7.71E+03	1.23E+04	1.13E+04
Fumaric acid	3.15E+05	2.88E+05	2.86E+05	2.99E+05	3.00E+05	2.76E+05
Cis-10-Heptadecenoic Acid	1.74E+05	1.61E+05	1.43E+05	1.62E+05	1.91E+05	1.61E+05
Citraconic acid	1.87E+05	1.68E+05	1.99E+05	1.38E+05	1.50E+05	1.58E+05
D-Arabitol	2.22E+05	1.96E+05	2.46E+05	9.58E+04	8.75E+04	1.01E+05
L-Arabitol	1.67E+05	1.65E+05	2.02E+05	7.41E+04	6.53E+04	8.54E+04
3-Aminosalicylic acid	7.66E+04	6.87E+04	6.27E+04	1.19E+05	1.26E+05	9.86E+04
Vanillin	8.68E+05	9.08E+05	1.05E+06	6.70E+05	8.66E+05	8.61E+05
2-Methylsuccinic acid	5.25E+06	4.89E+06	5.77E+06	3.65E+06	3.61E+06	4.11E+06
Benzoylformic acid	2.26E+05	3.03E+05	2.79E+05	1.58E+05	1.75E+05	1.79E+05
Phenethylamine	4.72E+06	5.71E+06	5.29E+06	4.84E+06	5.47E+06	5.96E+06
L-(-)-3-Phenyllactic acid	1.89E+05	2.03E+05	1.78E+05	1.76E+05	1.61E+05	1.81E+05
4-Guanidinobutyric acid	6.21E+05	6.20E+05	6.16E+05	9.07E+05	1.21E+06	8.09E+05
5-Methylcytosine	9.20E+04	9.05E+04	9.46E+04	7.20E+04	7.04E+04	6.17E+04
3-Hydroxybutyrate	1.45E+05	1.17E+05	1.33E+05	1.13E+05	1.22E+05	1.00E+05
Guanosine 3',5'-cyclic monophosphate	5.48E+06	5.71E+06	5.93E+06	1.77E+07	1.66E+07	2.12E+07
4-Hydroxybenzaldehyde	1.49E+06	1.42E+06	1.43E+06	1.35E+06	1.43E+06	1.52E+06
2,3-Dihydroxybenzoic Acid	3.97E+06	4.11E+06	3.99E+06	5.09E+06	5.49E+06	5.53E+06
1,7-Dimethylxanthine	3.07E+05	3.14E+05	2.69E+05	4.46E+05	2.87E+05	3.74E+05
Xanthosine	4.55E+05	3.75E+05	3.84E+05	4.64E+05	5.79E+05	5.09E+05
O-Phosphorylethanolamine	2.89E+05	2.67E+05	2.79E+05	1.54E+05	1.44E+05	1.59E+05

N-Propionylglycine	5.76E+04	7.13E+04	7.15E+04	5.84E+04	3.60E+04	5.93E+04
1-Caffeoylquinic acid	1.52E+07	1.46E+07	1.27E+07	1.79E+07	2.85E+07	1.63E+07
4-Hydroxybenzoic acid	3.69E+05	3.40E+05	3.45E+05	4.71E+05	5.24E+05	4.32E+05
Undecylic Acid	9.90E+05	6.11E+05	6.95E+05	6.61E+05	7.68E+05	7.61E+05
5-Oxoproline	1.18E+04	1.09E+04	1.38E+04	1.63E+04	1.31E+04	1.42E+04
Procyanidin B1	6.66E+05	6.18E+05	6.24E+05	5.73E+05	5.62E+05	5.37E+05
1-Methyladenine	1.58E+05	1.34E+05	1.39E+05	1.13E+05	1.03E+05	1.18E+05
Sodium Valproate	2.75E+06	2.40E+06	2.40E+06	2.85E+06	2.76E+06	2.69E+06
Spiraeoside	1.57E+06	1.52E+06	1.46E+06	2.28E+06	2.86E+06	2.28E+06
D-Glucose 6-phosphate	2.34E+06	2.33E+06	2.39E+06	1.52E+06	1.64E+06	1.71E+06
3’-Aenylic acid	3.77E+04	3.38E+04	4.65E+04	4.06E+04	4.26E+04	5.49E+04
Cyclic AMP	1.05E+05	9.33E+04	8.77E+04	2.43E+05	2.64E+05	2.80E+05
D-Serine	1.28E+04	1.74E+04	1.74E+04	1.63E+04	1.81E+04	1.71E+04
Coniferin	3.16E+06	4.08E+06	3.73E+06	2.46E+06	2.56E+06	2.47E+06
Trifolin	1.27E+07	1.12E+07	1.12E+07	1.20E+07	1.35E+07	1.40E+07
Pinobanksin	1.68E+06	1.45E+06	1.72E+06	2.09E+06	1.90E+06	2.60E+06
Kaempferin	9.23E+05	3.37E+05	3.13E+05	2.42E+06	3.68E+05	9.87E+05
Tricetin	1.13E+06	1.23E+06	8.90E+05	1.56E+06	1.80E+06	1.39E+06
p-Coumaryl alcohol	3.64E+04	2.51E+04	3.44E+04	3.11E+04	3.10E+04	4.83E+04
2-Methylglutaric acid	4.29E+05	4.20E+05	4.40E+05	5.01E+05	6.66E+05	5.38E+05
1,3,7-Trimethyluric acid	1.62E+04	1.73E+04	1.66E+04	2.57E+04	1.60E+04	2.24E+04
5-Hydroxyhexanoic acid	3.32E+04	4.09E+04	3.64E+04	6.08E+04	7.45E+04	7.11E+04
N-Oleoylethanolamine	3.98E+04	3.07E+04	3.38E+04	6.17E+04	6.88E+04	5.89E+04
Coumarin	8.63E+03	4.91E+03	5.12E+03	1.65E+04	2.16E+04	1.25E+04
Esculetin	2.22E+04	3.40E+04	2.37E+04	9.13E+04	1.01E+05	8.17E+04
Fraxetin	2.06E+05	2.30E+05	1.86E+05	2.47E+05	2.25E+05	1.90E+05

Esculin(6,7-DihydroxyCoumarin-6-glucoside)	3.84E+05	3.41E+05	2.68E+05	4.99E+05	5.01E+05	4.53E+05
Cyanidin 3-O-galactoside	6.84E+05	8.11E+05	8.07E+05	2.71E+06	1.60E+06	1.14E+06
Kaempferol	5.28E+05	5.13E+05	5.66E+05	6.19E+05	6.40E+05	6.36E+05
Apigenin 6,8-C-diglucoside	4.34E+05	4.16E+05	3.98E+05	9.47E+05	1.56E+06	1.13E+06
7-Methoxycoumarin	2.90E+05	3.53E+05	3.41E+05	5.61E+05	3.42E+05	2.20E+05
Galactinol	6.62E+06	5.21E+06	5.63E+06	1.01E+07	9.50E+06	9.99E+06
Glucose-1-phosphate	2.25E+06	2.55E+06	2.49E+06	1.58E+06	1.70E+06	1.66E+06
Batanin	8.32E+05	1.70E+06	1.09E+06	1.11E+06	9.56E+05	1.26E+06
Naringenin chalcone(4,2',4',6'-Tetrahydroxychalcone)	2.07E+06	1.76E+06	2.16E+06	2.57E+06	2.35E+06	3.17E+06
Garbanzol	1.10E+05	1.36E+05	9.72E+04	1.74E+05	8.78E+04	1.06E+05
D-Galacturonic acid(Gal A)	6.10E+04	5.26E+04	6.52E+04	5.99E+04	5.55E+04	6.17E+04
Methyl p-coumarate	5.18E+05	5.90E+05	6.80E+05	4.19E+05	4.46E+05	5.81E+05
Trans-4-Hydroxycinnamic Acid Methyl Ester	1.33E+06	1.38E+06	1.79E+06	8.59E+05	9.93E+05	1.27E+06
Tiliroside	1.55E+07	1.95E+07	1.65E+07	1.20E+07	1.82E+07	1.41E+07
Isoschaftoside	2.72E+06	1.94E+06	1.81E+06	4.91E+06	6.47E+06	4.28E+06
Theaflavin	5.12E+05	5.43E+05	6.00E+05	1.28E+06	1.34E+06	9.73E+05
Theaflavin-3-gallate	3.23E+05	3.32E+05	3.26E+05	5.23E+05	7.94E+05	6.32E+05
Theaflavin-3'-Gallate	2.42E+05	2.50E+05	2.89E+05	5.32E+05	4.87E+05	5.09E+05
Theaflavin 3,3'-Digallate	1.85E+06	2.45E+06	2.18E+06	2.41E+06	2.46E+06	2.98E+06
Orientin	4.81E+05	4.08E+05	3.45E+05	7.25E+05	9.73E+05	7.26E+05
Gossypitrin	1.83E+06	1.29E+06	1.47E+06	2.24E+06	2.74E+06	2.32E+06
Melibiose	7.35E+05	6.25E+05	8.24E+05	1.18E+06	1.09E+06	9.50E+05
D-Pantothenic Acid	2.20E+06	2.11E+06	2.09E+06	2.66E+06	2.32E+06	2.26E+06

Trans-ferulic acid	1.88E+05	2.16E+05	2.04E+05	3.52E+05	4.22E+05	3.27E+05
(-)-Epicatechin gallate	1.11E+08	1.08E+08	1.10E+08	1.00E+08	9.99E+07	9.77E+07
L-theanine	1.10E+06	1.06E+06	1.11E+06	1.13E+06	1.08E+06	1.03E+06
Indole-3-carboxylic acid	5.99E+04	7.52E+04	5.58E+04	7.60E+04	6.09E+04	8.09E+04
(-)-Epiafzelechin	2.36E+06	2.03E+06	2.36E+06	3.09E+06	3.22E+06	3.17E+06
Isovitexin	3.38E+05	3.36E+05	2.02E+05	1.03E+06	1.38E+06	5.96E+05
Stearic Acid	1.79E+07	1.38E+07	1.38E+07	1.77E+07	2.13E+07	1.74E+07
Eriocitrin	2.23E+05	1.88E+05	1.52E+05	2.32E+05	2.65E+05	2.86E+05
Salicin	1.88E+05	1.87E+05	2.37E+05	1.11E+05	8.64E+04	1.11E+05
Cynarin	1.94E+05	2.22E+05	1.61E+05	2.40E+05	3.18E+05	2.68E+05
Schaftoside	7.14E+03	7.19E+03	2.00E+03	1.30E+04	1.99E+04	4.96E+03
Diosmin	5.43E+04	5.23E+04	4.36E+04	5.24E+04	5.40E+04	7.98E+04
D-Pinitol	5.23E+04	5.40E+04	5.47E+04	6.58E+04	6.55E+04	6.29E+04
Cryptochlorogenic acid	8.98E+06	9.46E+06	7.70E+06	9.72E+06	1.12E+07	1.06E+07
Phloretin 2'-O-glucoside	1.03E+05	8.59E+04	9.14E+04	1.09E+05	1.02E+05	4.96E+05
Brevifolin?carboxylic acid	4.56E+06	5.84E+06	7.31E+06	3.65E+06	4.63E+06	4.64E+06
Astragalin	1.57E+07	1.28E+07	1.22E+07	1.57E+07	1.65E+07	1.47E+07
Caffeic acid	2.15E+05	2.11E+05	2.21E+05	4.29E+05	5.47E+05	4.89E+05
Cinnamic acid	1.05E+05	1.15E+05	1.10E+05	1.14E+05	1.16E+05	1.20E+05
Caffeine	3.23E+06	2.94E+06	3.08E+06	3.40E+06	3.00E+06	3.17E+06
Gallocatechin 3-O-gallate	4.72E+06	5.27E+06	5.30E+06	4.72E+06	4.52E+06	3.81E+06
Catechin gallate	1.68E+06	1.79E+06	1.62E+06	1.58E+06	1.56E+06	1.57E+06
Tyrosol	2.70E+04	2.41E+04	2.02E+04	3.01E+04	2.68E+04	3.22E+04
Matairesinol	1.22E+05	1.40E+05	1.65E+05	1.75E+05	1.08E+05	1.64E+05
Ursolic acid	5.90E+04	1.10E+05	1.12E+05	4.24E+04	1.66E+04	2.31E+04
1-Methoxyphaseollin	1.79E+04	1.70E+04	1.70E+04	2.35E+04	2.89E+04	1.80E+04

Oxidized Glutathione	1.82E+05	2.21E+05	2.22E+05	1.06E+05	1.17E+05	8.17E+04
D-Glucose	6.93E+05	6.92E+05	7.16E+05	1.18E+06	9.65E+05	1.21E+06
Isomaltulose	8.40E+05	1.00E+06	8.41E+05	1.33E+06	1.28E+06	1.31E+06
Esculin Hydrate	2.02E+05	1.68E+05	2.03E+05	2.87E+05	2.63E+05	2.26E+05
Turanose	1.54E+04	1.10E+04	6.75E+03	2.54E+04	2.11E+04	2.04E+04
Glycylphenylalanine	6.65E+05	6.11E+05	6.60E+05	1.14E+06	1.07E+06	1.19E+06
12-Hydroxydodecanoic acid	1.00E+04	1.02E+04	9.17E+03	1.39E+04	1.40E+04	1.25E+04
MAG(18:4)isomer1	2.28E+05	2.18E+05	2.26E+05	1.48E+05	1.94E+05	1.61E+05
MAG(18:3)isomer5	4.85E+05	4.24E+05	6.63E+05	1.10E+06	1.18E+06	9.20E+05
PC 16:1/14:1	3.57E+06	3.73E+06	3.36E+06	2.15E+06	1.65E+06	2.20E+06
MAG(18:4)isomer2	7.41E+04	7.12E+04	7.37E+04	6.53E+04	3.68E+04	5.61E+04
MAG(18:1)isomer2	1.56E+05	1.35E+05	1.44E+05	2.04E+05	2.92E+05	1.76E+05
Aminopurine	8.25E+05	8.13E+05	8.83E+05	2.10E+06	2.04E+06	2.33E+06
Hydroxy-methoxycinnamate	1.23E+05	1.42E+05	1.11E+05	7.84E+04	7.13E+04	8.69E+04
Choline	1.13E+07	1.15E+07	9.98E+06	1.76E+07	2.00E+07	1.78E+07
N',N'',N'''-p-Coumaroyl-cinnamoyl-caffeoyl spermidine	3.57E+05	2.94E+05	3.13E+05	2.85E+05	2.45E+05	2.15E+05
Nicotinic acid adenine dinucleotide	8.22E+04	8.71E+04	9.70E+04	1.13E+05	8.31E+04	8.00E+04
Cyanidin 3-O-glucoside(Kuromanin)	5.52E+05	7.05E+05	6.78E+05	2.21E+06	1.42E+06	8.10E+05
C-Hexosyl-luteolin O-hexoside	2.98E+04	3.91E+04	1.94E+04	6.07E+04	6.56E+04	3.71E+04
C-Hexosyl-apigenin O-pentoside	7.61E+04	6.07E+04	3.58E+04	1.85E+05	2.11E+05	7.16E+04
Di-C,C-hexosyl-apigenin	2.02E+06	2.91E+06	9.61E+05	5.65E+06	5.51E+06	2.19E+06
Luteolin 8-C-hexosyl-O-hexoside	1.96E+06	1.94E+06	1.60E+06	2.36E+06	2.22E+06	1.96E+06
Apigenin 8-C-pentoside	2.60E+05	2.17E+05	1.14E+05	3.52E+05	5.42E+05	3.33E+05
Tricin 7-O-hexoside	1.26E+06	9.39E+05	1.13E+06	2.14E+06	3.55E+06	2.06E+06
3-O-Feruloyl quinic acid	1.36E+05	2.17E+05	1.80E+05	2.97E+05	4.45E+05	2.46E+05

4-Methyl-5-thiazoleethanol	3.52E+04	3.90E+04	5.00E+04	3.09E+04	3.17E+04	3.86E+04
D-erythro-Dihydrosphingosine	6.44E+04	7.89E+04	7.22E+04	5.61E+04	6.41E+04	7.28E+04
LysoPC 18:3	1.88E+05	1.85E+05	1.91E+05	1.47E+05	1.87E+05	1.90E+05
LysoPC 16:0	8.55E+06	9.29E+06	8.55E+06	8.41E+06	8.89E+06	1.04E+07
LysoPE 18:1(2n isomer)	1.38E+06	1.33E+06	1.47E+06	2.69E+06	2.67E+06	2.23E+06
LysoPC 16:2(2n isomer)	1.48E+05	1.53E+05	1.64E+05	1.99E+05	2.00E+05	2.52E+05
LysoPE 14:0	4.67E+04	6.04E+04	4.71E+04	5.77E+04	5.68E+04	8.47E+04
LysoPC 18:3(2n isomer)	2.52E+06	2.54E+06	2.84E+06	3.41E+06	3.69E+06	3.64E+06
LysoPE 18:2(2n isomer)	1.96E+06	1.67E+06	1.86E+06	2.02E+06	2.22E+06	2.04E+06
LysoPE 16:0	9.67E+06	1.08E+07	1.05E+07	1.23E+07	1.26E+07	1.43E+07
Punicic acid	3.71E+06	3.54E+06	3.29E+06	3.56E+06	3.78E+06	3.66E+06
MAG(18:2)	4.58E+05	4.11E+05	4.65E+05	6.88E+05	5.89E+05	6.88E+05
Adenosine 5'-monophosphate	8.42E+05	1.39E+06	1.18E+06	2.07E+06	1.18E+06	2.03E+06
Indole	8.46E+05	7.83E+05	7.84E+05	9.14E+05	1.00E+06	9.45E+05
MAG(18:4)isomer3	3.29E+05	3.47E+05	3.12E+05	1.60E+05	1.68E+05	1.91E+05
4-Hydroxy-3,5-diisopropylbenzaldehyde	6.34E+04	6.00E+04	5.36E+04	9.38E+04	1.13E+05	7.47E+04
MAG(18:3)isomer3	4.06E+06	3.93E+06	3.75E+06	6.65E+06	5.46E+06	4.37E+06
MAG(18:3)isomer4	3.02E+04	2.59E+04	2.95E+04	4.95E+04	4.03E+04	5.26E+04
LysoPC 15:1	2.06E+04	1.95E+04	2.01E+04	2.57E+04	3.64E+04	3.62E+04
LysoPC 15:0	2.28E+05	2.87E+05	2.47E+05	2.52E+05	2.72E+05	2.71E+05
MAG(18:1)isomer1	4.90E+04	4.86E+04	4.79E+04	1.04E+05	1.08E+05	7.97E+04
LysoPC 17:0	1.55E+05	1.78E+05	1.51E+05	1.53E+05	1.45E+05	1.64E+05
MAG(18:3)isomer1	4.02E+06	3.78E+06	4.49E+06	4.59E+06	5.24E+06	5.49E+06
Acetyltryptophan	3.37E+06	3.73E+06	2.98E+06	2.66E+06	3.61E+06	2.90E+06
3,4-Dimethoxycinnamic acid	9.20E+04	1.21E+05	1.36E+05	3.67E+04	4.07E+04	8.52E+04
Lauric acid	6.64E+03	5.41E+03	5.33E+03	5.71E+03	6.36E+03	6.35E+03

Myristoleic acid	2.27E+03	1.56E+03	2.38E+03	1.21E+03	1.24E+03	1.99E+03
9,10-EODE	6.22E+06	6.44E+06	6.06E+06	7.12E+06	8.16E+06	8.34E+06
9-HOTrE	6.35E+05	6.48E+05	6.07E+05	6.31E+05	6.77E+05	7.10E+05
13-HOTrE(r)	6.26E+04	3.46E+04	4.95E+04	2.95E+04	4.25E+04	7.60E+04
12,13-EODE	4.53E+04	4.08E+04	4.00E+04	5.04E+04	5.12E+04	5.46E+04
2,5-Dihydroxy benzoic acid O-hexside	4.54E+06	4.42E+06	4.06E+06	3.44E+06	2.05E+06	4.19E+06
Uridine 5'-diphospho-D-glucose	2.83E+05	2.81E+05	2.85E+05	2.73E+05	3.43E+05	2.79E+05
Catechin-catechin-catechin	8.67E+05	8.33E+05	8.20E+05	6.08E+05	5.23E+05	8.03E+05
Cyanidin O-syringic acid	1.45E+04	2.83E+04	1.95E+04	5.81E+04	3.86E+04	2.25E+04
Acacetin-O-glucuronic acid	1.41E+05	1.15E+05	9.85E+04	1.98E+05	1.58E+05	1.06E+05
Apigenin 7-O-glucoside(Cosmosiin)	3.87E+04	3.39E+04	2.71E+04	4.08E+04	3.70E+04	4.91E+04
Eriodictyol C-hexoside	1.49E+05	9.95E+04	9.52E+04	2.87E+05	3.26E+05	1.95E+05
Luteolin C-hexoside	9.87E+04	8.27E+04	7.67E+04	2.16E+05	2.10E+05	1.23E+05
Quercetin O-acetylhexoside	1.97E+05	3.36E+05	1.49E+05	2.31E+05	2.12E+05	2.96E+05
Tricin O-saccharic acid	2.59E+05	3.34E+05	3.11E+05	3.22E+05	4.06E+05	5.38E+05
3-O-p-coumaroyl quinic acid O-hexoside	1.26E+05	1.43E+05	1.62E+05	1.40E+05	1.19E+05	2.15E+05
3-O-p-Coumaroyl quinic acid	2.42E+07	2.46E+07	2.50E+07	2.97E+07	3.29E+07	2.24E+07
Glucarate O-Phosphoric acid	4.82E+05	5.06E+05	4.12E+05	6.98E+05	8.48E+05	7.46E+05
Diethyl phosphate	2.14E+05	1.68E+05	1.37E+05	1.96E+05	1.82E+05	1.81E+05
Epicatechin-epiafzelechin	9.62E+04	9.84E+04	8.04E+04	1.19E+05	1.01E+05	1.27E+05
Di-O-methylquercetin	3.06E+05	3.08E+05	3.14E+05	5.53E+05	6.46E+05	5.97E+05
LysoPC 14:0(2n isomer)	5.44E+04	5.85E+04	4.44E+04	6.67E+04	7.28E+04	9.06E+04
LysoPC 16:0(2n isomer)	8.22E+06	8.77E+06	8.25E+06	9.36E+06	1.03E+07	1.15E+07
LysoPC 18:0	3.61E+05	3.74E+05	3.23E+05	4.32E+05	5.05E+05	4.81E+05
LysoPE 16:0(2n isomer)	2.20E+06	2.47E+06	2.20E+06	2.38E+06	2.89E+06	3.12E+06
L-Proline	2.43E+06	2.48E+06	1.96E+06	3.55E+06	3.27E+06	3.14E+06

L-Citrulline	3.79E+05	4.02E+05	3.73E+05	4.17E+05	4.32E+05	4.11E+05
L-(+)-Lysine	3.31E+06	3.90E+06	3.41E+06	4.71E+06	4.54E+06	4.35E+06
Adenine	5.21E+06	5.31E+06	5.29E+06	1.14E+07	1.01E+07	1.25E+07
Luteolin	1.94E+05	1.63E+05	1.40E+05	4.49E+05	4.63E+05	4.41E+05
2-Hydroxy-6-aminopurine	4.05E+04	4.51E+04	4.03E+04	4.36E+04	5.06E+04	3.95E+04
L-Glutamine	3.63E+06	4.14E+06	3.67E+06	5.40E+06	4.81E+06	4.30E+06
Adenosine	6.98E+06	6.79E+06	6.87E+06	1.51E+07	1.51E+07	1.80E+07
Benzoic acid	2.32E+04	1.61E+04	1.56E+04	1.87E+04	2.61E+04	2.44E+04
Xanthine	2.45E+04	2.73E+04	3.35E+04	4.13E+04	3.42E+04	4.13E+04
Uracil	6.01E+04	6.12E+04	5.35E+04	5.50E+04	6.33E+04	4.98E+04
Thymidine	7.87E+05	7.03E+05	5.99E+05	7.65E+05	7.10E+05	7.89E+05
Terephthalic acid	1.59E+06	1.55E+06	1.38E+06	1.39E+06	1.14E+06	1.55E+06
Phthalic acid	1.71E+05	1.64E+05	1.39E+05	1.35E+05	1.25E+05	1.71E+05
4-Acetamidobutyric acid	1.48E+06	1.21E+06	1.31E+06	7.97E+05	8.77E+05	6.85E+05
Methyl gallate	5.75E+07	6.03E+07	5.82E+07	4.15E+07	4.03E+07	4.21E+07
Kaempferol 7-O-rhamnoside	7.61E+05	2.90E+05	2.64E+05	1.81E+06	2.83E+05	7.64E+05
Apigenin 7-rutinoside(Isorhoifolin)	4.35E+05	2.19E+05	9.27E+05	5.01E+05	2.84E+05	1.62E+05
Kaempferol 3-O-rutinoside(Nicotiflorin)	2.09E+07	1.94E+07	1.73E+07	2.23E+07	2.60E+07	2.67E+07
Naringenin	1.68E+06	1.54E+06	1.79E+06	2.16E+06	1.92E+06	2.71E+06
Procyanidin B2	1.71E+07	1.45E+07	1.50E+07	1.38E+07	1.35E+07	1.64E+07
Malvidin 3-O-galactoside	3.64E+04	2.22E+04	2.38E+04	2.29E+04	2.24E+04	1.87E+04
L-Epicatechin	2.89E+06	3.08E+06	3.31E+06	4.29E+06	4.50E+06	4.44E+06
Xylitol	1.42E+05	1.41E+05	1.59E+05	8.87E+04	7.95E+04	9.18E+04
Inositol	3.47E+05	2.94E+05	3.58E+05	4.31E+05	4.38E+05	4.13E+05
D-(+)-Sucrose	3.09E+06	2.75E+06	3.11E+06	4.99E+06	4.30E+06	5.41E+06

Gluconic acid	3.07E+05	3.24E+05	3.41E+05	3.63E+05	3.02E+05	3.33E+05
L-Tyramine	3.89E+06	4.27E+06	5.38E+06	3.11E+06	4.74E+06	5.41E+06
Guanine	2.00E+05	2.41E+05	2.09E+05	1.79E+05	1.21E+05	1.42E+05
Allopurinol	2.41E+04	2.23E+04	2.21E+04	3.21E+04	3.29E+04	2.96E+04
Guanosine	1.62E+07	1.42E+07	1.56E+07	3.72E+07	4.04E+07	4.58E+07
Phloretin	5.08E+04	4.74E+04	4.42E+04	3.95E+04	3.94E+04	9.85E+04
3-Methylxanthine	9.50E+06	1.01E+07	8.97E+06	1.05E+07	9.08E+06	8.43E+06
Pyridoxine	6.60E+05	5.88E+05	6.45E+05	6.80E+05	6.30E+05	6.26E+05
Delphinidin 3-O-glucoside(Mirtillin)	2.09E+05	3.11E+05	2.13E+05	2.80E+05	3.76E+05	1.52E+05
p-Coumaric acid	9.45E+04	8.31E+04	8.14E+04	1.02E+05	8.04E+04	7.38E+04
5'-Deoxy-5'-(methylthio)adenosine	2.18E+06	2.37E+06	2.08E+06	1.99E+06	2.42E+06	1.87E+06
Kaempferol 3-O-robinobioside(Biorobin)	1.65E+07	1.67E+07	1.47E+07	2.09E+07	2.25E+07	2.39E+07
Isohemiphloin	1.32E+06	1.12E+06	1.06E+06	1.47E+06	1.43E+06	1.56E+06
Neochlorogenic acid(5-O-Caffeoylquinic acid)	8.05E+05	8.71E+05	7.41E+05	1.09E+06	1.45E+06	1.31E+06
DL-Arabinose	2.74E+05	2.75E+05	3.03E+05	2.44E+05	2.29E+05	2.60E+05
Dulcitol	2.87E+05	2.89E+05	3.52E+05	1.30E+05	8.40E+04	1.19E+05
Kaempferol 3,7-dirhamnoside(Kaempferitrin)	1.61E+05	1.55E+05	2.05E+05	1.78E+05	2.05E+05	1.93E+05
4-Pyridoxic acid	6.81E+04	6.78E+04	6.29E+04	7.27E+04	5.80E+04	6.66E+04
DL-Norvaline	3.72E+07	3.99E+07	3.80E+07	5.14E+07	5.11E+07	4.25E+07
4-Hydroxy-L-glutamic acid	3.25E+05	3.26E+05	2.80E+05	2.81E+05	2.14E+05	2.49E+05
7-Methylxanthine	2.04E+06	2.11E+06	1.97E+06	1.88E+06	1.74E+06	1.83E+06
L-Homocystine	1.36E+05	1.34E+05	1.62E+05	4.12E+05	5.51E+05	5.94E+05
3-Hydroxy-3-methylpentane-1,5-dioic acid	2.90E+05	2.75E+05	2.96E+05	2.40E+05	3.19E+05	2.57E+05

Quercetin	6.48E+05	4.53E+05	4.82E+05	8.21E+05	5.98E+05	7.84E+05
Trans-Citridic acid	4.64E+04	4.84E+04	5.62E+04	5.48E+04	4.72E+04	4.31E+04
2-Aminoisobutyric acid	1.18E+06	1.05E+06	9.51E+05	1.79E+06	2.24E+06	1.83E+06
N,N-Dimethylglycine	1.56E+05	1.50E+05	1.23E+05	2.75E+05	3.24E+05	2.74E+05
2'-Deoxyadenosine-5'-monophosphate	2.97E+04	1.99E+04	2.58E+04	6.44E+04	3.31E+04	2.60E+04
Uridine 5'-monophosphate	4.65E+05	4.06E+05	5.35E+05	1.09E+06	1.01E+06	8.89E+05
Trans,trans-Muconic acid	3.07E+05	3.92E+05	2.92E+05	3.21E+05	4.07E+05	3.55E+05
Quercetin 3-O-glucoside(Isotrifoliin)	1.72E+06	1.66E+06	1.61E+06	2.60E+06	3.33E+06	2.72E+06
Afzelechin(3,5,7,4'-Tetrahydroxyflavan)	1.11E+05	9.92E+04	1.17E+05	9.99E+04	4.88E+04	9.03E+04
N6-Succinyl Adenosine	6.32E+05	6.95E+05	8.22E+05	6.02E+05	5.33E+05	5.47E+05
H-HomoArg-OH	7.82E+04	7.05E+04	7.00E+04	1.01E+05	1.06E+05	1.04E+05
Sissotrin	9.14E+04	5.72E+04	8.77E+04	1.36E+05	1.60E+05	1.41E+05
Butin	1.05E+06	1.04E+06	1.09E+06	1.42E+06	1.25E+06	1.65E+06
D-Glucoronic acid	6.46E+04	5.74E+04	7.56E+04	7.15E+04	6.61E+04	6.81E+04
Cytidine	3.14E+05	3.16E+05	3.40E+05	7.80E+05	7.34E+05	6.47E+05
Deoxyadenosine	7.63E+05	7.84E+05	8.35E+05	4.93E+06	5.91E+06	4.54E+06
2-(Dimethylamino)guanosine	1.76E+06	1.65E+06	2.00E+06	2.20E+06	2.55E+06	2.00E+06
7-Methylguanine	1.12E+05	1.22E+05	1.09E+05	1.38E+05	1.17E+05	1.28E+05
2,6-Dimethyl-7-octene-2,3,6-triol	1.93E+05	1.85E+05	2.06E+05	1.90E+05	2.39E+05	2.05E+05
Oxalic acid	7.83E+04	1.03E+05	9.40E+04	1.29E+05	1.81E+05	8.77E+04
Protocatechuic acid-4-glucoside	6.47E+06	6.65E+06	5.80E+06	5.15E+06	4.03E+06	6.04E+06
1-Hydroxyterpinin monoglucoside	2.10E+04	2.43E+04	3.13E+04	2.96E+04	3.50E+04	2.89E+04
Terpineol monoglucoside	1.67E+06	1.79E+06	2.03E+06	2.50E+06	3.19E+06	2.22E+06
Isochlorogenic acid A	3.81E+06	5.34E+06	3.89E+06	4.56E+06	5.24E+06	3.43E+06
Isochlorogenic acid C	3.98E+06	4.74E+06	4.78E+06	5.04E+06	5.12E+06	3.81E+06

3-O-(E)-p-Coumaroyl quinic acid	1.58E+07	1.64E+07	1.62E+07	1.91E+07	2.14E+07	1.87E+07
Galloylgallic acid	5.93E+06	6.43E+06	9.17E+06	3.89E+06	6.06E+06	5.20E+06
3-Galloylshikimic acid	1.14E+05	1.19E+05	1.28E+05	5.29E+04	4.90E+04	4.88E+04
5-Galloylshikimic acid	2.33E+05	3.42E+05	2.95E+05	1.62E+05	8.83E+04	1.56E+05
Galloyl Methyl gallate	1.09E+07	1.34E+07	1.97E+07	4.07E+06	4.89E+06	6.62E+06
"Tetragallic Acid(3""-O-galloyl-4""-O-galloyl-4-O-galloyl-galloyl acid)"	1.56E+05	1.83E+05	2.05E+05	7.18E+04	8.21E+04	8.00E+04
Tri-O-Galloylshikimic acid	1.71E+05	1.67E+05	2.02E+05	3.20E+05	5.68E+05	2.31E+05
Methyl 5-galloyl gallate	1.17E+05	8.84E+04	1.06E+05	1.02E+05	7.95E+04	1.07E+05
Gentisic acid	5.26E+06	5.52E+06	4.98E+06	6.45E+06	7.32E+06	7.10E+06
Hexadecanoic acid	4.90E+06	5.61E+06	4.20E+06	4.09E+06	4.76E+06	4.14E+06
Eicosadienoic acid	4.41E+04	4.28E+04	4.68E+04	5.41E+04	6.72E+04	5.25E+04
Hexahydroxydiphenoyl galloylglucose	1.89E+05	1.51E+05	3.41E+05	1.23E+05	1.61E+05	1.16E+05
Glucogallin	9.80E+06	9.62E+06	1.03E+07	6.30E+06	8.88E+06	5.49E+06
Hexahydroxydiphenoylglucose	1.27E+06	1.86E+06	2.19E+06	9.76E+05	1.35E+06	1.44E+06
Tetragalloylglucose	1.84E+06	2.27E+06	2.28E+06	7.86E+05	7.67E+05	8.54E+05
Cynaroside	4.27E+06	2.28E+06	1.41E+06	9.19E+06	1.23E+06	3.81E+06
Procyanidin C1	4.46E+05	3.44E+05	3.34E+05	5.23E+05	3.36E+05	4.42E+05
Cinnamtannin B2	7.37E+04	6.49E+04	3.75E+04	5.40E+04	7.02E+04	6.85E+04
Syringaresinol	1.99E+04	2.46E+04	2.64E+04	6.13E+04	3.86E+04	3.25E+04
Procyanidin B4	8.79E+05	6.95E+05	6.78E+05	7.90E+05	7.07E+05	7.81E+05
10,16-Dihydroxy-palmitic acid	6.78E+04	5.75E+04	6.60E+04	1.97E+05	2.49E+05	1.45E+05
9S-Hyroxy-10E,12E-octadecadienoic acid	1.61E+06	1.63E+06	1.52E+06	1.72E+06	1.76E+06	1.94E+06
9-Hydroxy-12-oxo-10-octadecenoic acid	3.04E+04	3.23E+04	3.30E+04	3.46E+04	4.16E+04	4.36E+04
9,12,13-Trihyroxy-10,15-octadecadienoic acid	6.32E+04	6.02E+04	6.53E+04	1.34E+05	1.70E+05	1.52E+05

9,10,13-Trihyroxy-11-octadecadienoic acid	2.99E+05	3.04E+05	3.16E+05	5.64E+05	6.41E+05	6.47E+05
Trihydroxycinnamoylquinic acid	1.56E+05	1.41E+05	1.59E+05	1.42E+05	2.23E+05	1.68E+05
Quillaic acid	8.80E+05	9.62E+05	1.60E+06	4.79E+05	1.60E+05	4.20E+05
Hispidulin	5.68E+05	2.55E+05	3.54E+05	7.17E+05	6.73E+05	7.64E+05
Jaceosidin	2.23E+05	1.87E+05	1.67E+05	3.88E+05	5.03E+05	3.38E+05
5-Hydroxy-6,7,3',4'-tetramethoxyflavone	4.85E+06	4.32E+06	4.33E+06	4.11E+06	2.86E+06	2.94E+06
Apigenin-8-C-glucoside	1.64E+06	1.16E+06	7.62E+05	2.89E+06	2.96E+06	1.95E+06
Luteolin-6,8-di-C-glucoside	4.73E+04	2.81E+04	3.77E+04	8.29E+04	9.03E+04	6.34E+04
Trans-3-O-p-coumaric quinic acid	4.71E+06	4.44E+06	4.74E+06	6.25E+06	7.30E+06	7.14E+06
Cis-3-p-coumaric quinic acid	4.84E+06	5.36E+06	5.77E+06	7.53E+06	1.09E+07	7.61E+06
Apigenin-6-C-glucose-8-xylcose	1.52E+05	1.53E+05	9.05E+04	2.46E+05	4.32E+05	1.66E+05
Apigenin-6-C-2-glucuronylxyloside	2.51E+05	3.26E+05	2.05E+05	5.23E+05	5.45E+05	2.23E+05
Procyanidin B3	2.95E+05	2.45E+05	3.14E+05	2.59E+05	1.78E+05	2.42E+05
Procyanidin C2	3.50E+05	2.76E+05	3.20E+05	3.42E+05	4.31E+05	3.34E+05
Chlorogenic Acid	9.28E+06	9.59E+06	8.50E+06	9.31E+06	1.15E+07	1.02E+07
Pratensein	5.34E+05	2.83E+05	3.61E+05	6.70E+05	7.45E+05	9.20E+05
Pinocembrin	2.93E+04	3.01E+04	3.61E+04	3.12E+04	2.87E+04	5.55E+04
Apigenin	3.84E+04	4.17E+04	3.37E+04	8.99E+04	9.53E+04	1.03E+05
Acacetin-7-O-galactoside	9.89E+04	7.25E+04	8.94E+04	1.64E+05	1.92E+05	1.62E+05
Diosmetin-7-O-galactoside	4.08E+05	3.54E+05	3.71E+05	6.92E+05	7.80E+05	6.72E+05
Luteolin-7-O-rutinoside	1.96E+07	2.56E+07	2.68E+07	2.76E+07	3.15E+07	3.06E+07
Matairesinoside	1.93E+05	2.01E+05	3.06E+05	2.47E+05	1.59E+05	2.15E+05
Isoorientin	4.89E+05	3.89E+05	3.06E+05	8.09E+05	6.88E+05	4.53E+05
Lonicerin	1.35E+06	2.01E+06	1.58E+06	1.93E+06	2.13E+06	2.25E+06

Vitexin-2-O-D-glucopyranoside	1.23E+05	9.88E+04	4.77E+04	2.56E+05	3.75E+05	1.70E+05
6-Deoxyfagomine	8.89E+05	1.07E+06	7.67E+05	1.50E+06	1.05E+06	9.14E+05
Phenylalanine	7.37E+06	6.72E+06	6.05E+06	8.27E+06	8.76E+06	8.37E+06
PC(18:2)	1.14E+07	1.19E+07	1.24E+07	1.05E+07	1.28E+07	1.12E+07
PC(18:2)isomer	9.47E+05	9.89E+05	9.54E+05	1.15E+06	1.13E+06	1.29E+06
5,8,11,14-Pentadecanoamide	3.66E+04	3.27E+04	2.19E+04	4.45E+04	3.68E+04	5.27E+04
Hexadecylsphingosine	9.14E+06	9.04E+06	7.79E+06	1.06E+07	1.17E+07	1.07E+07
LysoPC(16:2)	2.29E+06	2.38E+06	2.34E+06	2.53E+06	2.96E+06	3.04E+06
2,3-Dihydroxypropyl-9,12-octadecadienoate-hexose-hexose	5.32E+04	5.73E+04	6.48E+04	7.40E+04	8.66E+04	5.62E+04
3-{(2-Aminoethoxy)(hydroxy)phosphoryl]oxy}-2-12-octadecadienoate	1.82E+05	1.79E+05	1.74E+05	1.40E+05	1.92E+05	1.83E+05
LysoPC(18:2)	1.20E+07	1.28E+07	1.21E+07	1.22E+07	1.31E+07	1.31E+07
3-Hydroxypropyl palmitate glc-glucosamine	1.53E+06	1.61E+06	1.61E+06	2.11E+06	3.01E+06	2.34E+06
2,3-Dihydroxypropyl-9,12,15-octadecatrienoate-hexose-hexose	6.31E+05	6.48E+05	5.73E+05	9.04E+05	1.02E+06	9.21E+05
3-{(2-Aminoethoxy)(hydroxy)phosphoryl]oxy}-2-hydroxypropyl palmitate	1.06E+07	1.13E+07	1.09E+07	1.26E+07	1.22E+07	1.55E+07
LysoPC(16:1)	9.16E+06	9.95E+06	8.94E+06	9.67E+06	1.00E+07	1.11E+07
LysoPC(18:1)	9.88E+06	8.65E+06	1.04E+07	2.12E+07	2.19E+07	1.98E+07
Propyl2-(trimethylammonio)ethyl phosphate	3.12E+05	2.66E+05	3.20E+05	6.20E+05	8.40E+05	6.39E+05
Glycerin Monopalmitate	9.58E+03	8.21E+03	6.36E+03	9.10E+03	1.30E+04	1.00E+04

Phthalic anhydride	4.49E+05	4.16E+05	4.37E+05	1.05E+06	6.49E+05	4.91E+05
LysoPC(18:0)	1.74E+06	1.76E+06	1.61E+06	2.34E+06	2.31E+06	2.56E+06
6-Hydroxykaempferol-7-O-glucoside	6.29E+06	6.60E+06	7.46E+06	7.94E+06	7.25E+06	7.29E+06
6-Hydroxykaempferol-3,6-O-Diglucoside	2.65E+05	3.09E+05	2.13E+05	4.68E+05	4.60E+05	4.98E+05
6-Hydroxykaempferol-7,6-O-Diglucoside	5.00E+04	3.68E+04	3.58E+04	3.51E+04	3.67E+04	3.41E+04
