# Supplementary material for: Unpruning improvement the quality of tea through increasing the levels of amino acids and reducing contents of flavonoids and caffeine
Source: Front Nutr. 2022 Sep 29;9:1017693. doi: 10.3389/fnut.2022.1017693 (PMC9558131; doi:10.3389/fnut.2022.1017693)
Supplement: Supplementary file 4 [file Data_Sheet_3.PDF]

	A1	A2	A3	A4	A5	A6	B1
	1	1	1	1	1	1	2
Asp	0.018503	0.187618	0.183313	0.187374	0.01875	0.01822	0.15896
Glu	0.003013	0.001757	0.002425	0.002789	0.028776	0.019017	0.001583991
Ser	0.067225	0.05927	0.064142	0.056184	0.068835	0.061089	0.042116122
His	0.000986	0.000746	0.001084	0.000975	0.00087	0.000772	0.0004158
Gly	0.001432	0.000814	0.001009	0.000876	0.001005	0.000558	0.001219506
Thr	0.003514	0.002911	0.004949	0.005217	0.005455	0.003365	0.008776336
Ala	0.004459	0.000761	0.001138	0.001212	0.001176	0.004487	0.001462155
Theanine	9.75796	9.428687	8.882206	9.362777	10.195	11.26783	7.157824281
Arg	0.002113	0.002453	0.001828	0.002111	0.002306	0.002538	0.002449896
Tyr	0.000538	0.000582	0.000583	0.00055	0.000562	0.000584	0.000587898
Cys	0.003529	0.001932	0.003788	0.004011	0.004138	0.001975	0.003742559
GABA	9.78E-05	9.93E-05	7.59E-05	7.37E-05	9.96E-05	9.67E-05	0.000109749
Val	0.000334	0.011842	0.000393	0.000431	0.020767	0.014024	0.000501589
Met	0.018588	0.018187	0.018277	0.018476	0.018306	0.012156	0.000309948
Phe	0.018393	0.016588	0.019628	0.017562	0.016424	0.015945	0.000949194
Ile	0.014187	0.013864	0.01456	0.014517	0.014463	0.014041	0.007167118
Leu	0.005553	0.005698	0.006183	0.006376	0.006631	0.006384	0.001837328
Pro	0.000677	0.000738	0.000738	0.000878	0.007075	0.000736	0.000731921
GA	0.641741	0.623148	0.737408	0.730909	1.023173	1.003455	0.833401558
GC	7.5689	7.372148	8.773937	8.66985	13.4249	12.2808	7.331932921
Theophyll	0.244214	0.264871	0.302619	0.29917	0.557785	0.412716	0.515021164
EGC	3.428149	3.538295	4.176971	4.130151	5.972125	5.538444	4.699777612
C	2.856094	2.995646	3.571959	3.558135	4.975902	4.851235	4.677600983
CA	15.7059	16.02048	18.31198	18.14276	25.10993	25.02458	21.32972596
EGCG	13.83827	15.10497	18.23633	18.1474	25.89674	26.47402	20.10229553
EC	4.291168	4.415871	5.426999	5.336363	7.283763	7.424379	5.672288587
GCG	0.711549	0.782387	0.970325	0.902296	1.192202	1.267037	1.351775112
ECG	11.49991	12.43447	15.48707	15.44948	22.25767	22.73498	15.42499114
Taxifolin	0.072962	0.089057	0.115427	0.110269	0.059196	0.064953	0.062087049
CG	0.144468	0.152532	0.184062	0.182959	0.275041	0.27258	0.147188768
Rutin	1.265655	1.286533	1.563779	1.565429	2.31267	2.357232	1.383840211
Ellagic a	0.041849	0.049539	0.050167	0.055067	0.085831	0.086359	0.035633346
Myricetin	0.063651	0.053604	0.065857	0.072239	0.115887	0.111481	0.067581691
Luteolin	0.021113	0.02206	0.027458	0.027783	0.010349	0.010332	0.031827229
Kaempfero	0.011895	0.00957	0.010905	0.010303	0.014271	0.017295	0.011995076

B2	B3	B4	B5	B6
2	2	2	2	2
0.15374	0.015762238	0.014682008	0.017649251	0.015846069
0.000949422	0.025912208	0.023487134	0.029281976	0.026754759
0.03700449	0.059028856	0.052049565	0.05757186	0.042308942
0.000419385	0.000446712	0.000536152	0.00041137	0.000418147
0.001141556	0.001033561	0.000817889	0.001092926	0.001032228
0.005345996	0.008177646	0.007127303	0.008746721	0.008005865
0.001295319	0.00166212	0.001483699	0.001691271	0.001316506
4.223740766	7.152402376	6.298973871	7.757269842	6.574233921
0.001068598	0.002194235	0.002492894	0.00261179	0.002411044
0.000577903	0.00052878	0.00054976	0.00050481	0.00051216
0.003847351	0.002587269	0.002173946	0.004447542	0.002361569
7.38E-05	0.000117739	8.84E-05	0.000152938	0.000156393
0.000246426	0.000218168	0.000223767	0.027852799	0.024355316
0.000282291	0.020600678	0.018649476	0.017989991	0.019742316
0.000773044	0.012646105	0.009468535	0.012641206	0.01363398
0.004412567	0.004461054	0.004351162	0.000432246	0.00043398
0.000721178	0.007621267	0.006561396	0.008202953	0.00719744
0.000748593	0.001821702	0.001545522	0.00136601	0.0007349
0.836285421	0.98128153	0.984925564	0.989754248	1.007061763
7.394542539	8.640390307	8.692761172	8.603055658	8.730866568
0.537919208	0.63334773	0.623893883	0.657059349	0.641153116
4.677602502	5.388801127	5.441441819	5.629712786	5.681743494
4.826761373	5.349826918	5.354314507	5.368430518	5.452256141
21.27418607	24.80977654	24.94767465	24.80796716	25.17844562
21.58123195	26.25601647	26.28495885	26.11402906	26.56773015
5.731548346	6.842886045	6.832022414	6.769354794	6.874403904
1.39998484	1.61785399	1.58620276	1.633921938	1.661138608
16.41978893	20.04466569	20.0964176	19.77679452	20.0897115
0.053536906	0.067444167	0.076260476	0.074690408	0.073131203
0.157604723	0.186108237	0.193805646	0.191655339	0.189257092
1.40036048	1.707992963	1.711475135	1.703982117	1.723331744
0.051004823	0.065860475	0.066327274	0.068114793	0.06072392
0.069661252	0.094003743	0.094808278	0.093109706	0.074854584
0.009649378	0.012418003	0.012591301	0.012346737	0.013278103
0.010286833	0.013513257	0.013547183	0.012606346	0.014817868
